# Numerical and experimental studies on dynamic gas emission characteristics of boreholes

**DOI:** 10.1371/journal.pone.0251209

**Published:** 2021-05-07

**Authors:** Huaying Lin, Shixiang Tian, Anjun Jiao, Jianhua Zeng, Zebiao Jiang, Shiqing Xu, Xionggang Xie, Jun Tang

**Affiliations:** 1 College of Mining Engineering, Guizhou University, Guizhou, Guiyang, 550025, China; 2 The National Joint Engineering Laboratory for the Utilization of Dominant Mineral Resources in Karst Mountain Area, Guizhou University, Guizhou, Guiyang, 550025, China; 3 School of Safety Engineering, China University of Mining and Technology, Xuzhou, Jiangsu, 221116, China; University of New South Wales, AUSTRALIA

## Abstract

The gas emission rate of boreholes is one of the most important indices for coal and gas outburst prediction. In this work, instantaneous gas emission velocity and environmental effects on borehole gas emission were studied. Through theoretical analysis, the mechanism of crack propagation in the coal borehole was clarified, and the effect of soft and hard coal on gas desorption and gas emission. The results of numerical simulations also indicated that the initial gas emission has a function relationship to the drilling distance and the physical characteristics of the coal seam. A novel dynamic testing technology was proposed to obtain gas emission velocity. Laboratory experiments under adsorption and desorption of CO_2_ and N_2_ were performed using coal samples from Xuehu, Fenghui, Weishe, and Wuzhong coal mines. The data of initial gas emission under different coal samples were recorded, and the fitting curves were obtained. The results show a positive correlation between initial gas emission and drilling depth. However, abnormal would occur when the drill pipe enters the soft stratification, and the maximum value of the initial gas emission of the abnormal part is 3.8 times the normal value, which indicates a high degree of sensibility to soft stratification. The results were revealed the dynamic gas emission law of boreholes.

## 0 Introduction

Various accidents such as coal and gas outbursts, gas explosions and fire generally occur in coal mining [1–6]. In these accidents, coal and gas outbursts often cause the most serious damage [7–12]. Coal and gas outburst is closely related to the amount of gas emission at the initial moment after the coal destroyed by in situ stress. Thus, the analysis of the initial gas emission rate from boreholes has important engineering significance.

Many researchers have conducted adsorption-desorption experiments on coal samples with different particle sizes. It was concluded that as the particle size of a coal sample decreases, the adsorption equilibrium pressure increases, and the gas desorption rate becomes faster [13–19]. Through the analysis of coal adsorption pores, it is found that adsorption pores may have multi-modal pore size distribution (PSD) and the maceral composition mainly dominates the development of the specific surface area (SSA) and pore volume (PV), which in turn affects the gas adsorption capacity [20–24]. A number of scholars have researched on the law of coal seam gas flow. For instance, it shows that the maximum rate of gas output from drilling occurs a few seconds before the coal body is destroyed, and the gas diffusion rate of tectonic coal at the initial stage of diffusion is greater than that of the primary coal. The attenuation of the latter stage diffusion rate is greater than that of the primary coal. The tectonic coal stratification induces an increase in the plastic volume, and maximum plastic deformation of the adjacent primary coal through the interface stress, which promotes the release of gas inside the primary coal [25–27]. When a coal seam is damaged, its permeability changes simultaneously. Researches on coal seam gas show that the larger the permeability coefficient of the coal seam, the higher the maximum value of gas emission rate from its borehole [28,29]. The gas migration law in a coal seam is the basis for studying gas emission from a borehole. In the 1970s, Chinese researchers began to study the initial velocity of gas emission from boreholes. A mathematical model for the coal seam gas migration was established and was applied to calculate the initial velocity of gas emission from borehole [30–34]. Some other researchers proposed to consider factors, such as gas desorption and migration to simulate the initial velocity of gas emission from boreholes [35,36]. Research on gas emission from boreholes shows that the rate of velocity change is largest at the initial moment, and it increases with the gas adsorption pressure of coal seams [37].

Under the disturbance caused by drilling, coal fractures propagate within the in-situ stress-concentrated zones [38]. In these fractures and pores, gas desorbs fast and it is emitted quickly into the environment. In unmined coal seams, the gas remains relatively in its original state of occurrence, and coal gas adsorption and desorption are under dynamic equilibrium [39]. In drilling process, with a rapid increase in the stress and instantaneous change in the gas pressure around the surface of the borehole, the desorbed gas emits into the borehole constantly. Therefore, the amount of gas emission from the borehole varies with the drilling process.

However, previous studies focused on the gas emission model of exposure coal body, which simulates the effect of ground stress, gas pressure, and coal seam permeability on initial gas emission in a static state. The research on gas emission law of borehole has been mainly focussed on the later stage of drilling simultaneously and dynamic gas emission characteristics of borehole are rarely studied.

Therefore, in this paper the dynamic gas emission law of borehole was studied by theoretical analysis and numerical simulation. The effect of different coal type and different occurrence state on dynamic gas emission law of borehole is analyzed. Further, the laboratory experiment under determination of initial gas emission was also presented in this work and verify the accuracy of the numerical simulation.

## 1 Theoretical analysis of gas emission from borehole

### 1.1 Stress analysis of coal seam pore

The microstructure of the pores is usually unpredictable; however, the overall shape is oblate, and a stress concentration appears in the longitudinal section [40]. For the convenience of subsequent research on the evolution of the cracks, it is assumed that the pores are elliptical. The longitudinal stress σ_1_ is greater than the transverse stress σ_3_. The equation of static equilibrium of coal pore structure is show in Eq 1 [41].

$$\sigma '=\sigma -\sigma_{\text{p}}-\sigma_{sw}$$ (1)

Where σˊ represents the effective stress, MPa; σ indicates the in-situ stress on coal, MPa; σ_p_ denotes the pore pressure caused by gas, MPa; and σ_sw_ signifies the expansion stress caused by coal adsorption of gas, MPa.

**Fig 1:**
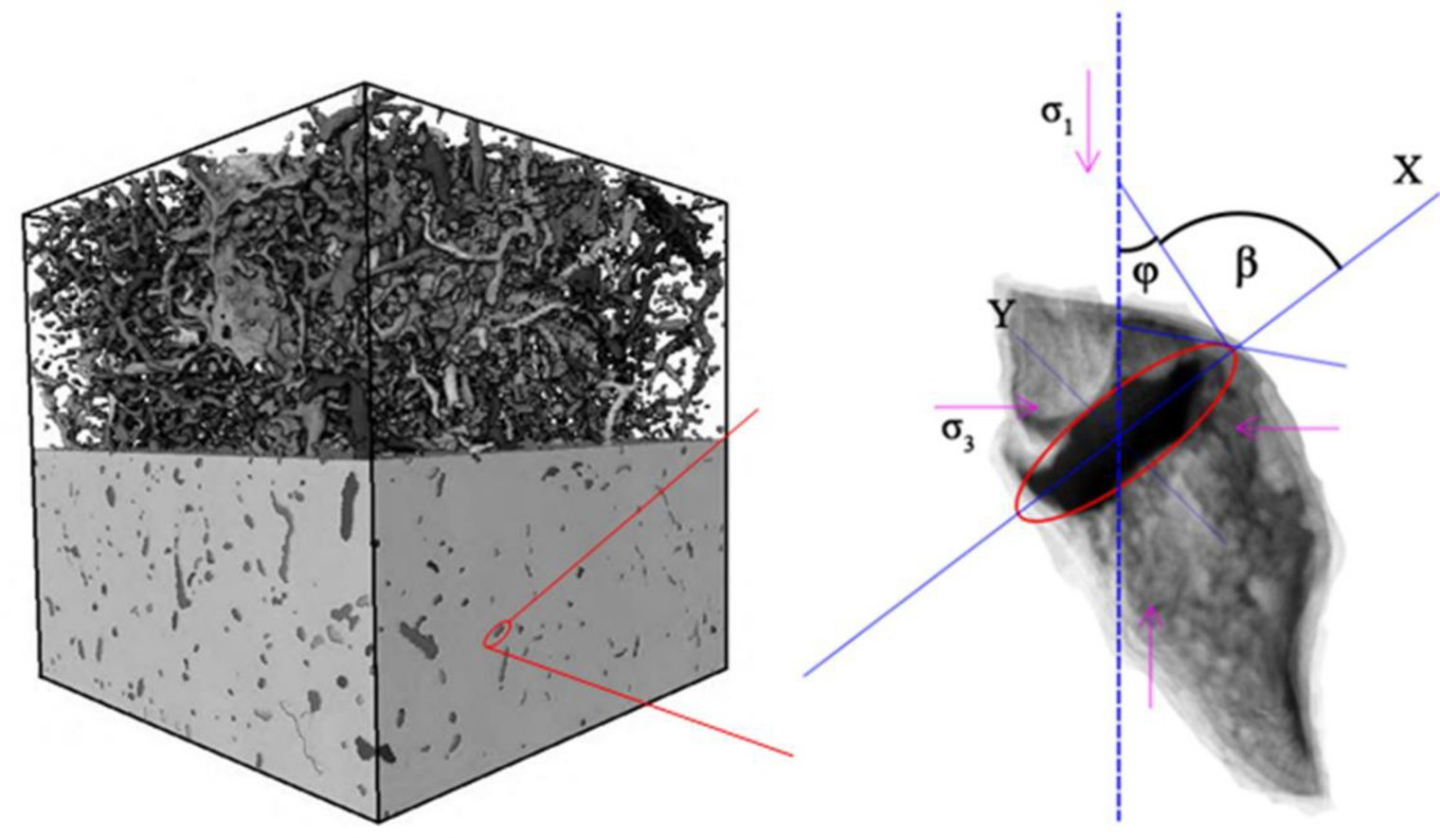
Schematic diagram of coal pore structure.

To facilitate the subsequent analysis, the coordinate system shown in Fig 1 is established, and adopting the Griffith criterion and introducing the uniaxial tensile strength σ_t_, the limit value of micro-crack failure, when the coal body is subjected to an effective stress can be expressed by the following equation:

$$\{\begin{array}{c} {(\sigma_{1}^{'}-\sigma_{3}^{'}}^{2}-8\sigma_{t}(\sigma_{1}^{'}+\sigma_{3}^{'})=0\\ \sigma_{3}^{'}=-\sigma_{t} \end{array}\;\begin{array}{c} (\sigma_{1}^{'}+3\sigma_{3}^{'})>0\\ (\sigma_{1}^{'}+3\sigma_{3}^{'})<0 \end{array}$$ (2)

Where σ_1_ˊ and σ_3_ˊ signifies the effective vertical and horizontal stresses.

The stress failure of coal is not caused by a single factor, but by the interaction among many factors. The Cartesian coordinates are transformed into polar coordinates, which are further simplified to a = c×ch*ζ* and b = *c*×ch*ζ*; then, the slope near the end of the elliptical fracture is

$$tg\delta =\frac{dy}{dx}=\frac{\varsigma_{0}}{\eta }$$ (3)

According to the above Eq 4, it possibility of a fracture is the greatest that when the included angle φ with σ_1_ˊ is 30 to 40°.

$$tg\delta =\frac{\varsigma_{0}}{\eta }=\frac{\tau xy}{\left(\sigma y\pm {\left(\sigma_{y}^{2}+\tau_{xy}^{2}\right)}^{0.5}\right)}=\frac{-\left(\sigma_{1}^{'}-\sigma_{3}^{'}\right)\sin2\varphi }{2\left(\sigma_{1}^{'}\cos^{2}\;\varphi +\sigma_{3}^{'}\sin^{2}\;\varphi \right)\pm {\left(\sigma_{1}^{'2}\cos^{2}\;\varphi +\sigma_{3}^{'2}\sin^{2}\;\varphi \right)}^{0.5}}$$ (4)

Where *δ* is the effective stress angle, °; *ζ*_0_ is the coordinate value of the elliptical crack; *η* is the coordinate value of the crack tip; *τ*_*xy*_ is the effective shear stress; and *φ* is the angle of easy cracking as well as the angle between the direction of rupture and the longitudinal axis.

According to the analysis of energy transmission and attenuation, when the tensile strength of the coal body cannot balance the external disturbance, the coal body starts deforming. As a result, new cracks are produced or original cracks are further expanded. Under the action of tectonism, the tensile strength of coal decreases, and the damage of coal is more serious under the same energy. And simultaneously, the pore system of coal becomes more developed, especially for the pores in the range of mesopores and macropores. The development of micron-sized pores makes the seepage process of gas easy and smooth in the coal. This change breaks the gas adsorption desorption equilibrium, leading to rapid gas desorption, and ultimately resulting in a sharp increase in coal desorption rate in the early stage of gas release [42].

### 1.2 Mathematical model

#### 1.2.1 Governing equation of gas transport

The two states of free state and adsorption state are the main forms of gas in coal, so the gas content of coal seam can be expressed as the sum of the two [43]:

$$\frac{\partial M}{\partial t}=\frac{\partial M_{x}}{\partial t}+\frac{\partial M_{y}}{\partial t}$$ (5)

Free state gas content *M*_*y*_ is:

$$M_{y}=\rho \varphi$$ (6)

$$\begin{array}{l} \frac{\partial M_{y}}{\partial t}=\beta \left(P\frac{\partial \varphi }{\partial t}+\varphi \frac{\partial P}{\partial t}\right)\\ =\beta \left(\frac{P(1-\varphi_{0})}{{\left(1+\varepsilon_{v}\right)}^{2}}\frac{\partial \varepsilon_{v}}{\partial t}+\frac{\varphi_{0}+\varepsilon_{v}}{1+\varepsilon_{v}}\frac{\partial P}{\partial t}\right) \end{array}$$ (7)

The adsorption state gas content *Mx* can be obtained by Langmuir Eq [44]:

$$M_{x}=\frac{abcP\rho_{n}}{1+bP}$$ (8)

Among them:

$$c=\rho_{s}\left(\frac{1}{1+0.147e^{0.022V_{ad}}}e^{n(T_{s}-T)}\right)\frac{100-M_{ad}-A_{ad}}{100}$$ (9)

Where *a* is the adsorption constant, coal adsorption limit, m^3^/t; *b* is the adsorption constant, MP^-1^; *c* is the coal calibration parameters, kg/m^3^; *ρ*_*n*_ is the gas density at standard atmospheric pressure, kg/m^3^; *ρ*_*s*_ is the density of coal, kg/m^3^; *T*_*s*_ is the the temperature of the adsorption experiment in the laboratory, °C; *T* is the underground coal body temperature, °C; *M*_*ad*_ is the moisture content in coal, %; *V*_*ad*_ is the volatile content in coal, %; *A*_*ad*_ is the ash content in coal, %.

Among them:

$$n=\frac{0.02}{0.993+0.07P}$$ (10)

$$\frac{\partial M_{x}}{\partial t}=\frac{abc\rho_{n}}{{\left(1+bP\right)}^{2}}\frac{\partial P}{\partial t}=\frac{\beta abcP_{n}}{{\left(1+bP\right)}^{2}}\frac{\partial P}{\partial t}$$ (11)

Substituting Eqs 7 and 11 into Eq 5, get:

$$\frac{\partial M}{\partial t}=\frac{\partial M_{x}}{\partial t}+\frac{\partial M_{y}}{\partial t}=\frac{\beta abcP_{n}}{{\left(1+bP\right)}^{2}}\frac{\partial P}{\partial t}=\beta \left(\frac{P(1-\varphi_{0})}{{\left(1+\varepsilon_{v}\right)}^{2}}\frac{\partial \varepsilon_{v}}{\partial t}+\frac{\varphi_{0}+\varepsilon_{v}}{1+\varepsilon_{v}}\frac{\partial P}{\partial t}\right)$$ (12)

The gas migration of coal body conforms to the law of conservation of mass, and the coal seam gas seepage conforms to Darcy’s law [45]. Combining the Eq 12, the gas migration control equation in the fluid-solid coupling model of the coal body around the borehole is derived:

$$\frac{2P(1-\varphi_{0})}{{\left(1+\varepsilon_{v}\right)}^{2}}\frac{\partial \varepsilon_{v}}{\partial t}+\frac{\varphi_{0}+\varepsilon_{v}}{P(1+\varepsilon_{v})}\frac{\partial P^{2}}{\partial t}+\frac{abcP_{n}}{{\left(1+bP\right)}^{2}}\frac{\partial P^{2}}{\partial t}=\Delta \cdot \left(\frac{K}{\mu }\cdot \Delta P^{2}\right)$$ (13)

#### 1.2.2 Dynamic evolution model of permeability

In the process of gas desorption and seepage, the effective stress effect has a negative adjustment effect on coal seam gas permeability with the decrease of gas pressure, and the matrix shrinkage effect has a positive adjustment effect on coal seam gas permeability with the decrease of gas pressure. Due to their joint action, the coal seam gas permeability is in a dynamic state [46,47].

Available according to the concept of porosity:

$$\varphi =\frac{V_{\varphi }}{V_{v}}=\frac{V_{\varphi_{0}}+\Delta V_{P}-\Delta V_{v}}{V_{v_{0}}-\Delta V_{v}}=\frac{\varphi_{0}+\Delta \varepsilon_{P}-\varepsilon_{v}}{1-\varepsilon_{v}}$$ (14)

Coal adsorption-desorption deformation is generally divided into two parts. One-third of the adsorption expansion deformation at the contact point is converted into adsorption expansion stress, which changes the effective stress and affects the overall deformation; while the two-thirds of the adsorption expansion deformation directly changes the volume of the crack, but it is not included in the deformation of the skeleton [48].

When the gas pressure drops from *P*_0_ to *P*:

$$\varepsilon_{v}=\frac{\Delta \sigma '}{K}=\frac{1}{K}(P_{0}-P+\sigma_{P_{0}}-\sigma_{P})=\frac{\Delta \sigma_{P}+\Delta P}{K}$$ (15)

Among them:

$$\varepsilon_{P}^{'}=\frac{2acT\rho_{v}{\text{R}}_{\text{m}}}{3K{\text{V}}_{\text{m}}}\text{ln}\left(\frac{1+bP_{0}}{1+bP}\right)$$ (16)

Change the effective stress, desorption shrinkage stress and matrix shrinkage:

$$\Delta \varepsilon_{P}=\frac{2}{3}\varepsilon_{P}^{'}\;\Delta \sigma_{p}=\frac{1}{3}\varepsilon_{P}^{'}$$ (17)

Substitution can get:

$$\varphi =\varphi_{0}+\frac{\left(2K+\varphi_{0}-1\right)}{1-\varepsilon_{v}}-\frac{2\Delta P}{1-\varepsilon_{v}}$$ (18)

According to the Kozeny-Carman formula, the permeability can be calculated:

$$k=k_{0}{\left(\frac{\varphi }{\varphi_{0}}\right)}^{3}=k_{0}{\left[1+\frac{(2K+\varphi_{0}-1)\varepsilon_{v}}{(1-\varepsilon_{v})\varphi_{0}}-\frac{2\Delta P}{(1-\varepsilon_{v})\varphi_{0}}\right]}^{3}$$ (19)

## 2 Numerical simulation

### 2.1 Model building

**Fig 2:**
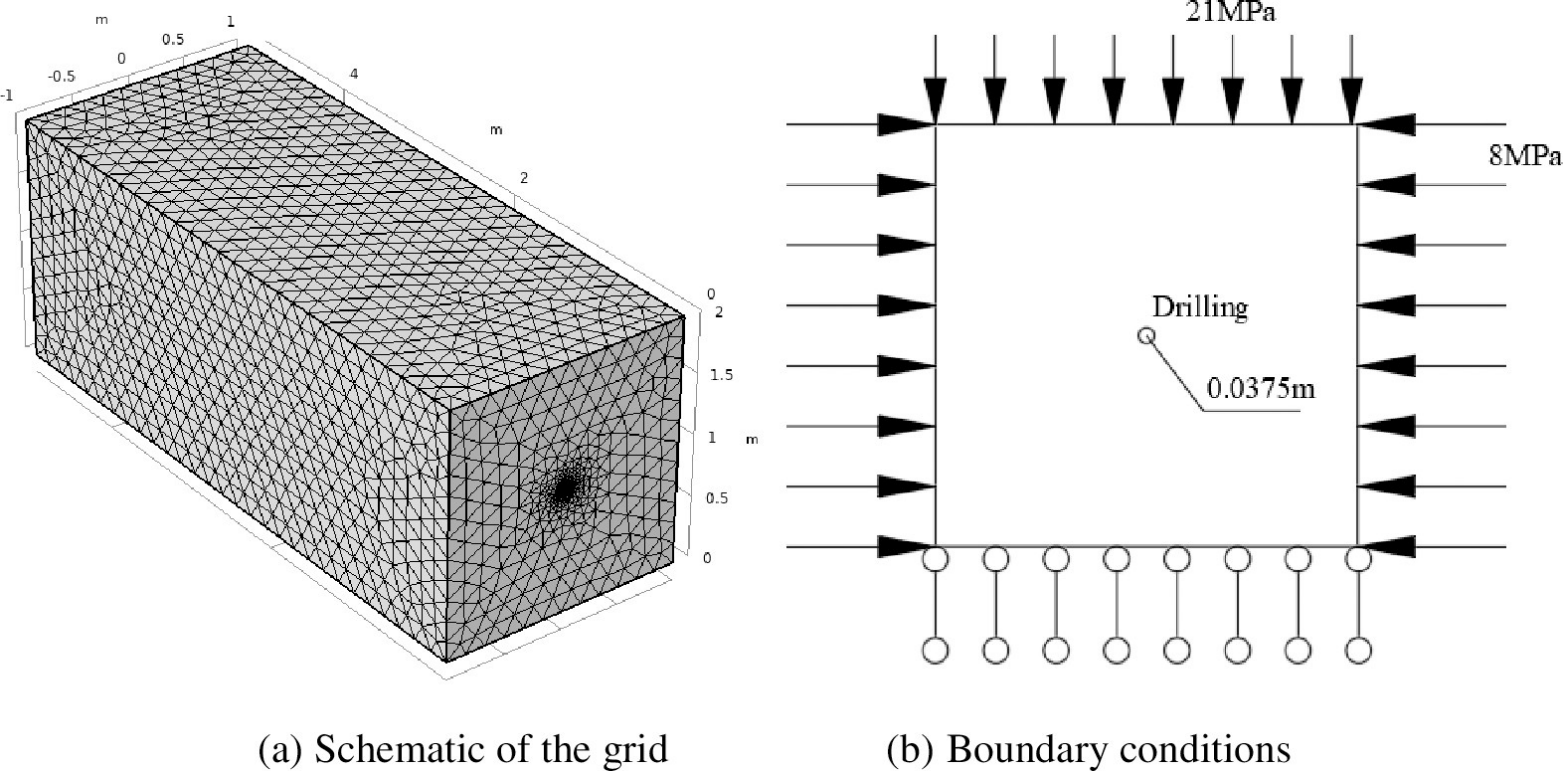
Simplified numerical model.

In this paper, the numerical simulation of COMSOL Multiphysics was used to analyze the initial gas emission in the borehole. The numerical physical coal model was built with a length of 5 m, and height and width of 2 m. The diameter of borehole was 0.0375 m. The grid around the hole and the bottom of the hole are subdivided to meet the calculation accuracy requirements, and the remaining parts are free tetrahedral grids with a minimum element size of 2 mm, (See Fig 2A). Considering the influence of the in-situ stress and other boundaries on the borehole, a longitudinal load of 21 MPa and a horizontal load of 8 MPa were applied to the model, (See Fig 2B). A drill pipe was drilled forward at a constant speed of 0.22 m/s and the drilling was stopped when the drilling distance reached 4 m.

To investigate the law of gas emission from the boreholes by the simulation, the model size and simulation plan were determined according to the phenomenon of gas migration and gas desorption. The mechanical parameters of soft and hard coal in 2104 haulage roadway of Xuehu Coal Mine were measured, and the values of numerical simulation parameters were referred to the measured results. The physical parameters of the model are determined by the coal sample under laboratory conditions, and shown in Table 1.

**Table 1:** Inputs to the model: basic parameters.

Type	Elasticity modulus (MPa)	Poisson’s ratio (–)	Porosity (–)	permeability (m^2^)	Gas density (kg/m^3^)	Kinetic viscosity (Pa·s)	Internal friction angel (°)	Cohesion (MPa)
strong coal	2713	0.29	0.03	2.33e–17	1350	1.85e–5	35	0.93
soft coal	673	0.45	0.09	1.5e–19	1350	1.85e–5	22	0.137

### 2.2 Analysis of borehole instantaneous gushing velocity

Due to new cracks are generated around for during the drilling process, the coal body stress is redistributed. The gas of fracture zone migrates to the borehole through the newly generated fracture network, and the decrease in the gas pressure causes the pressure of the coal body to vary regionally, relative to that of the primeval coal seam.

**Fig 3:**
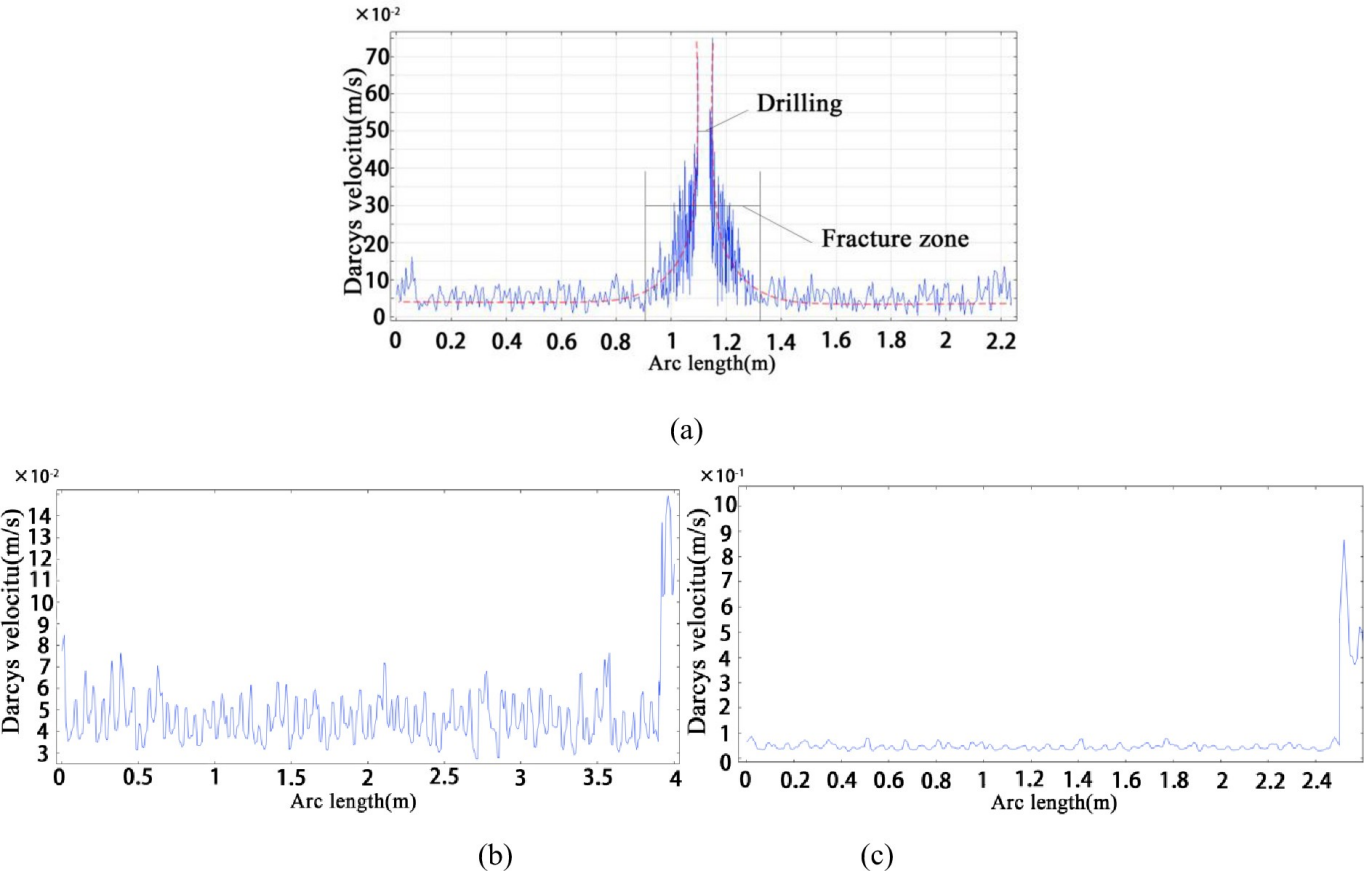
Initial gas emission rate of a borehole: (a) instantaneous gas emission velocity of a two-dimensional borehole; (b) instantaneous gas emission velocity of a hard coal-hole wall; (c) instantaneous gas emission velocity of a hard coal contains soft coal.

Based on the simulation results, it was observed that a pressure fracture zone was formed around the borehole. In the fracture zone as the energy of from the borehole to the coal wall decayed, the number and sizes of the cracks continued to decrease until they reached an equilibrium state. Owing to an increase the fracture aperture in the fracture zone, the permeability of the area increased, the coal body was desorbed and gas emission from the borehole increased sharply. The instantaneous gas emission velocity of borehole the fracture zone reached 0.75 m/s. The pressure gradient decreased with an increase in the borehole distance. Therefore, the amount of gas desorption was less and the gas flow rate was less than one-seven of the peak value. According to the change of gas emission velocity, the diameter of the fracture zone is 0.5 m, (See Fig 3A). Outside this area, the coal and rock contributed insignificantly to the gas emission. It can be seen from the instantaneous gas emission velocity curve of the hole wall that the gas desorption was concentrated 2 s before the coal body was exposed, and the instantaneous gas emission velocity reached 0.15 m/s. As the coal body exposure time increased, the instantaneous gas emission velocity decreased and tended to reach a state of dynamic equilibrium, and its gushing speed fluctuated slightly by 0.05 m/s, (See Fig 3B); The gas emission velocity of the area, where the drill bit passed through the tectonic coal was significantly higher than the normal velocity, and the instantaneous maximum value reached 0.89 m/s, which was nearly four times the normal level, as shown in Fig 3C.

### 2.3 Initial gas emission law of the borehole

**Fig 4:**
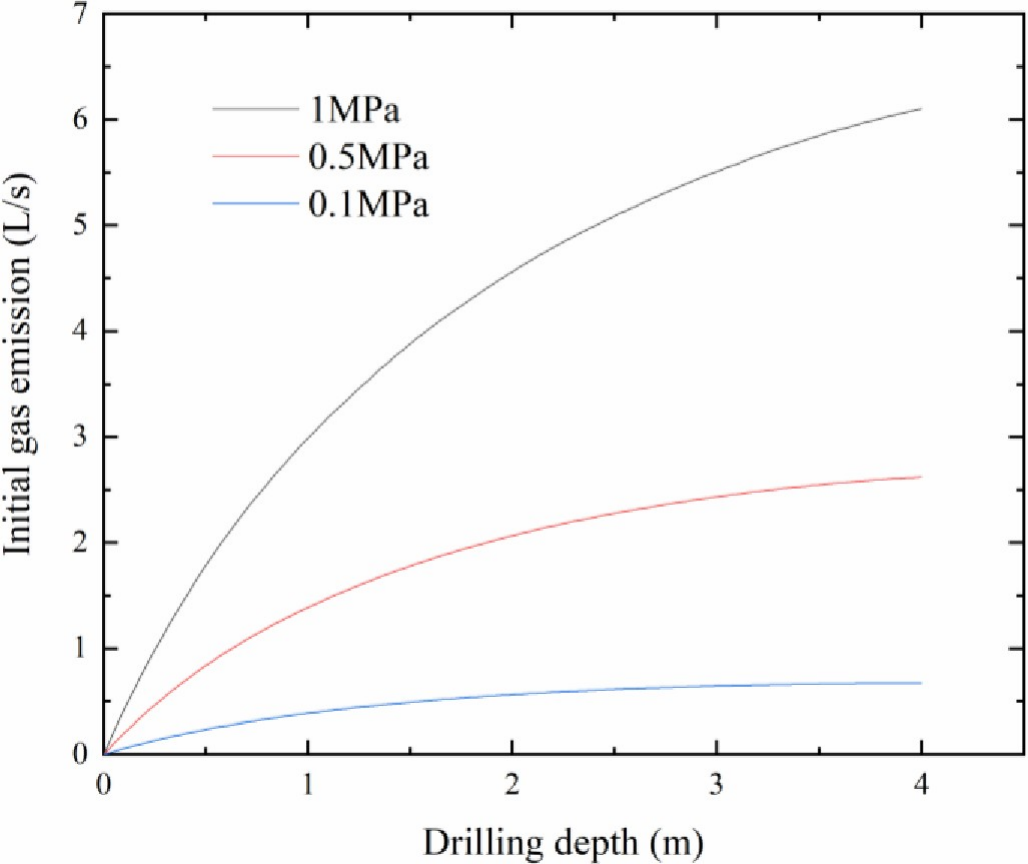
Drilling depth versus initial gas emission.

As shown in Fig 4, under each particular gas pressure, the initial gas emission increased rapidly with the drilling process. From the perspective of the whole drilling process, the increasing rate of initial gas emission decreased with an increase in the drilling depth. Furthermore, at the same drilling depth, compared with the initial gas emission under different gas pressures, we can find that there is a clearly evident positive correlation between the initial gas emission and gas pressure. As the gas pressure gradually increased, the initial gas emission curve increased accordingly.

As the release of gas from the coal body was not completed in a short time, the drill bit broke the original equilibrium state after contacting the coal body and the coal body was suddenly exposed to the environment; therefore, the external stress acting on the coal disappeared and the gas was quickly transformed from an adsorbed gas to the free state. After the coal body was exposed, the gas release was concentrated in the first few seconds, and with the passage of time, the gas desorption decreased. As the drill bit advanced further, new coal bodies were constantly exposed, and the gas emission from the previous coal bodies continuously increased, (See Fig 4).

Under the action of the geological processes, the occurrence conditions and physical properties of the coal body in the local area were different from those of the overall coal seam. To study the law of the initial gas emission, when the drill passed through the soft coal seam, a soft coal seam was set in the model with a thickness and depth of 1m and 2.5 m, respectively.

**Fig 5:**
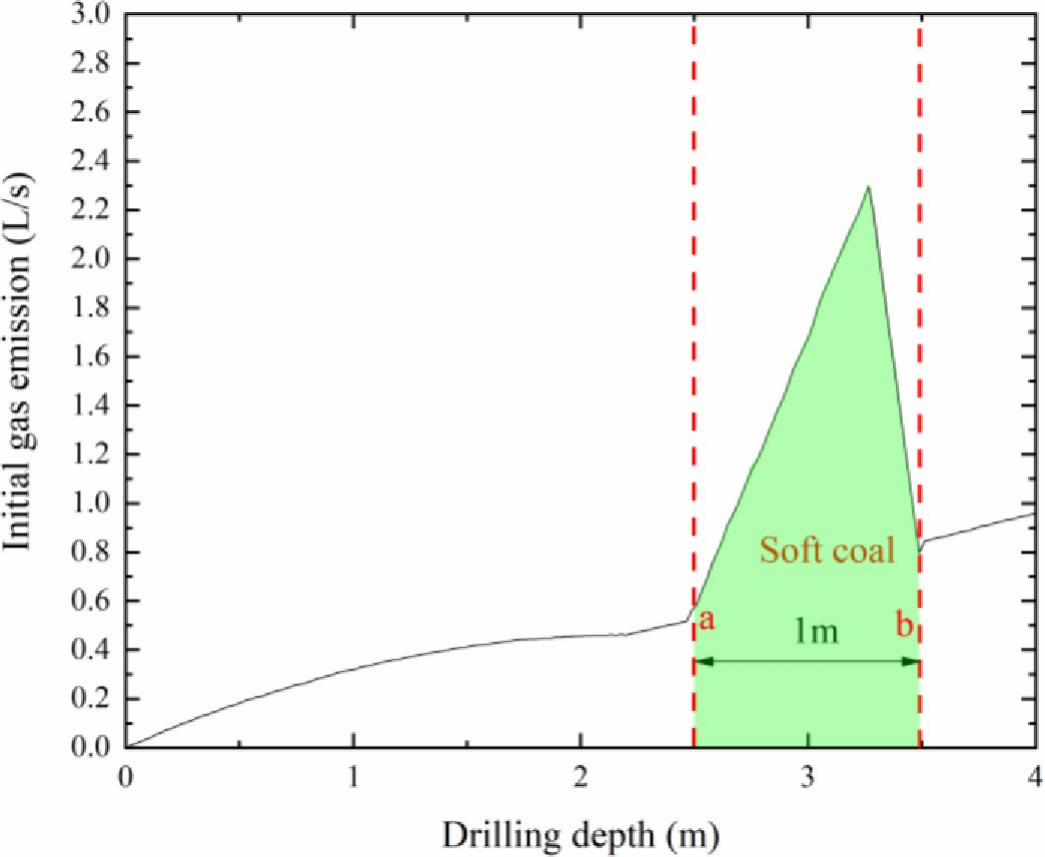
Local soft coal drilling depth versus initial gas emission.

As shown in Fig 5, there was a significant difference between the gas emissions of the borehole in the soft coal seam and the hard coal. Due to the permeability of the soft coal seam was reduced, the gas was more likely to accumulate in the soft coal seam. Under the same occurrence conditions, the amount of gas released by the soft coal was much greater than that of the hard coal; therefore, there was a sharp rise in the graph, as shown by point ‘a’. Most of the borehole gas emerged from the newly exposed coal, and the curve returned to the original trend after the borehole passed through the soft coal seam, as shown by point ‘b’.

According to the theoretical analysis and simulation presented above, it was found that the initial gas emission from the boreholes in the same type of coal seam was also significantly different. To further understand the influence of the crustal stress, coal seam water content, and initial gas pressure on the initial gas emission, which was emitted from the borehole were studied using the numerical model.

**Fig 6:**
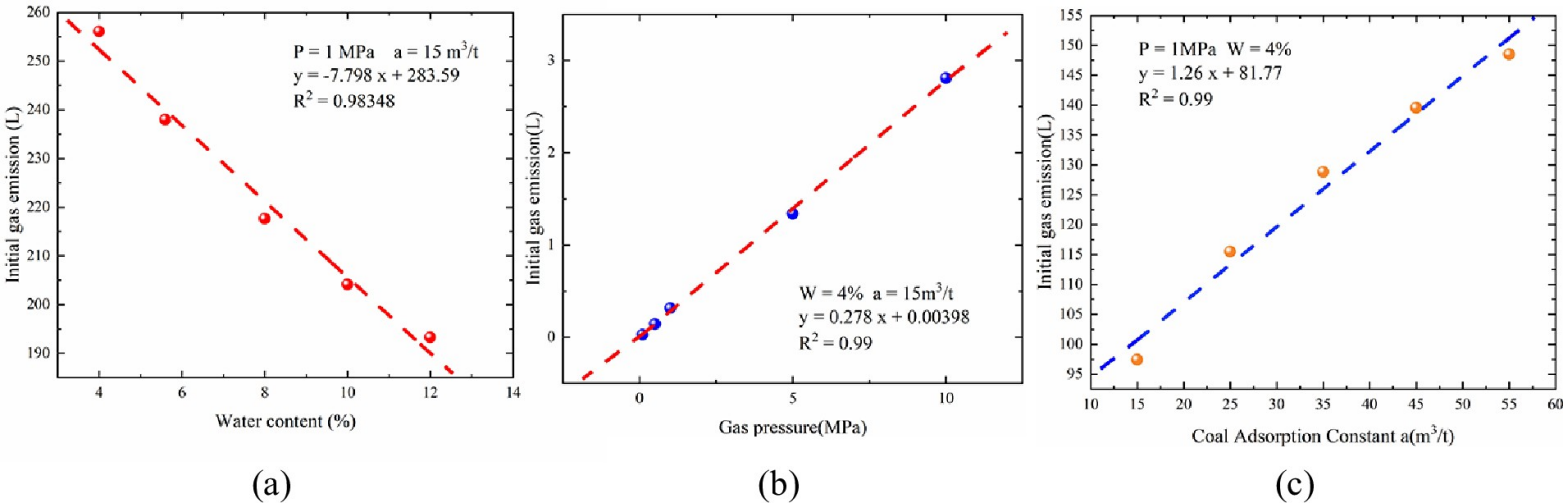
Initial gas emission versus coal physical properties: (a) water content versus initial gas emission; (b) gas pressure versus initial gas emission; (c) coal adsorption constant ‘a’ and initial gas emission.

The main forces between the coal and adsorbed molecules were van der Waals forces and hydrogen bonds. The surface of the coal adsorbed the water molecules. Because the hydrogen bond between the coal and water molecules was greater than the van der Waals force between the coal and methane molecules, the adsorption energy of methane molecules was significantly reduced, the adsorption equilibrium distance increased, and methane was adsorbed to unstable positions. Compared to the adsorption of methane, water molecules would be more easily adsorbed. The water molecules would be in an adsorption state, and methane molecules, in a desorption state, indicating that water was in a dominant position when molecules of water and methane participated in competitive adsorption. Therefore, the higher the moisture content of the coal, the lower the amount of gas adsorbed by the coal seam, and the lower the initial gas emission from the borehole when it was damaged (See Fig 6A). The coal seam gas pressure could be used to evaluate the amount of gas stored in the coal seam. The gas pressure was directly proportional to the amount of gas adsorption. The greater the pressure, the higher the content. On the other hand, the higher the gas pressure inside the coal seam, the greater its potential energy. When the borehole damaged the coal body, when the pressure was released, the surrounding coal and rock were subjected to three forces: gas potential energy, drill bit impact, and strength of the coal body. The higher the coal body gas pressure, the more serious the damage to the coal body would be. The larger the radius of the fracture zone, the larger the gas emission, and hence, the larger the amount of initial gas emitted (See Fig 6B). The coal seam gas adsorption can be expressed in terms of the coal’s gas adsorption constant ‘a’ and ‘b’. The larger the value of adsorption constant ‘a’, the greater the amount of gas stored in the coal, and greater the amount of gas desorbed when damaged by external forces (See Fig 6C).

## 3 Experimental procedure

### 3.1 Sample preparation and test process

**Fig 7:**
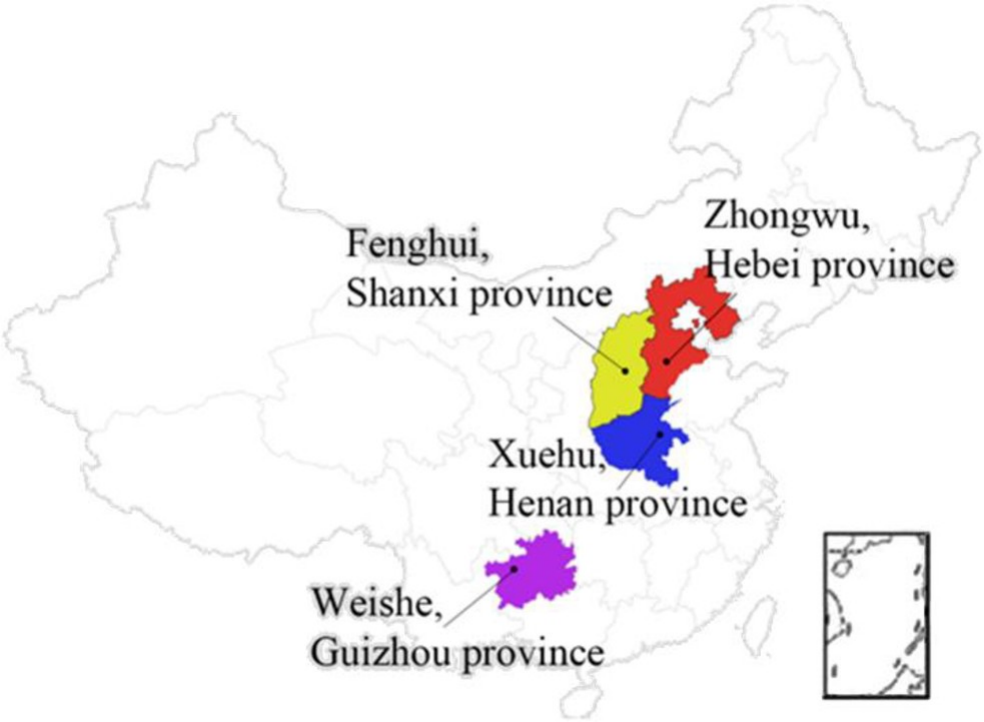
Distribution map of coal specimen sampling sites.

The specimens used in this study to carry out the initial gas emission experiment were collected from four different coal-mining areas. They were (i) No. 9 coal seam of Wuzhong Coal Mine, (ii) No. 2 coal seam of Xuehu Coal Mine, (iii) No. 15 coal seam of Fenghui Coal Mine, and (iv) M29 coal seam of Weishe Coal Mine (See Fig 7).

**Fig 8:**
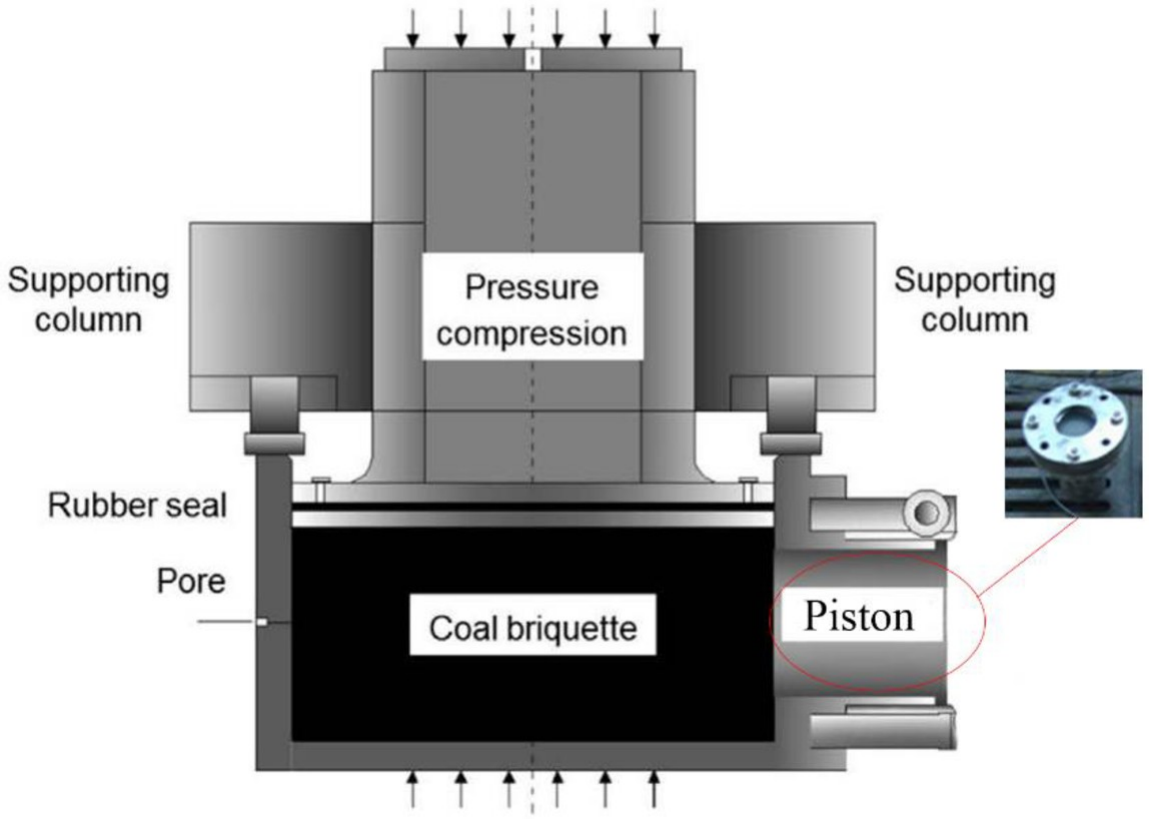
Coal seam simulation device.

During the test, a 42-mm twist drill was used. The coal sample was selected and pressed into a briquette, and loaded into a cylinder with a height of 330 mm. The upper part of the cylinder was pressured by a YF-10000F press (See Fig 8). The piston in the front of the cylinder was filled with Portland cement to simulate the surrounding rock and white latex is applied to the surface after the cement had solidified and passed the air tightness test.

**Fig 9:**
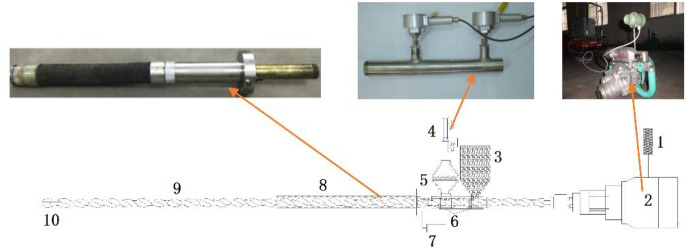
Schematic diagram of the “linear continuous predictive” device used for the prediction of coal roadway outburst. 1 –displacement sensor; 2 –coal electric drill; 3 –funnel for coal cuttings; 4 –flow velocity sensor; 5 –screen; 6 –three-way; 7 –manual pressure test pump; 8 –borehole sealing equipment; 9 –twisted drill pipe; and 10 –twisted drill bit.

Experiment used the “linear continuous predictive” device, (See Fig 9). The twist drill pipe comprised three parts: the first 1080 mm was the length of sleeve; the last 60 mm was the length of broken coal; and the remaining part was the sampling part of initial gas emission data.

Through a 12-h vacuum extraction, a vacuum condition could be ensured in the coal chamber, the gas was filled into the cylinder 24 hours before the experiment, and the load was applied to assist the gas migration. And it was loaded with an axial force of 100 KN. The loading stress is divided into three stages. The first loading is about 2% of the predetermined value, the second loading is 50% of the predetermined value, and the third loading is to the predetermined value. By pumping CO_2_ or N_2_ into the coal, the coal was sealed under 100 KN of pressure force and was kept pumped for approximately 48 h to reach the adsorption and desorption equilibrium. Next, the gas pressure and loading stress of the coal chamber was checked in advance to continue the experiment. The experimental test distance was 1 m. The flow meter was pushed at a constant speed and the data were automatically read by the main machine every 0.5 s. The data were gathered up to 30 s after the end of the drilling.

### 3.2 Proximate analysis of coal sample

The moisture (Mad), ash (Aad), volatile components (Vdaf), initial rate of gas emission (Δp), coal adsorption constant (a and b) of the coal samples were measured, and the measurement results are shown in Table 2.

**Table 2:** Physical parameters of coal samples.

Mine	Proximate analysis			△p (mm Hg)	a (m^3^/t)	b (MPa^-1^)	Type
	Mad (%)	Aad (%)	Vadf (%)				
Xuehu	4.73	9.65	9.97	11.4	26.18	1.72	Lean coal/Anthracite
Fenghui	3.54	23.33	7.37	22.6	31.25	0.68	Anthracite
Wuzhong	7.4	14.26	22.22	11.5	30.21	2.11	Coking coal
Weishe	5.62	10.71	10.49	24.1	28.58	1.43	Anthracite

## 4 Results and discussion

**Fig 10:**
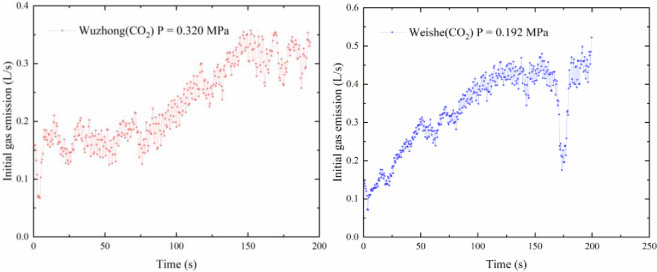
Initial gas emission versus drilling depth.

The initial gas emission was tested with coal samples from different coal mines. To facilitate the recording of the drilling length, the test used a constant speed drilling machine and the recorded time was used to replace the drilling distance. The relationship between the gas emission and time is shown in Fig 10.

**Fig 11:**
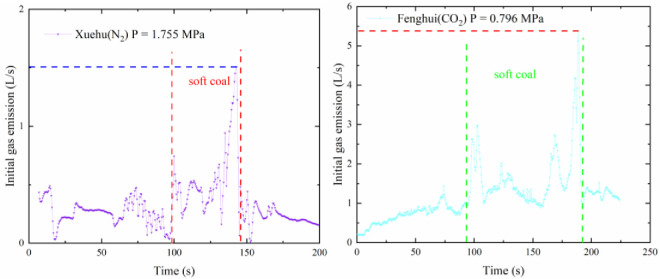
Initial gas gushing amount from the borehole of the local soft coal seam.

The samples were prepared by collecting soft coal seams from the coal mines, to test the gas emission experiments from boreholes on hard coal contains soft coal. The coal samples preparation was carried out smaller particle-size pulverized coal, and placed in the middle of the cylinder block to simulate the initial gas emission on hard coal contains soft coal. In this case, the gas emission data are shown in Fig 11.

Compared with the initial gas emission data of Xuehu Coal Mine, the curve was 0.5 L/s in the normal area, and the peak gas flow rate reaches 1.5 L/s when the borehole enters the soft coal. In the numerical simulation stage, the peak value of the normal area of borehole gas was 0.85 L/s, and the peak value of soft coal was 2.2 L/s. The abnormal gas area in the experimental part is 3 times higher than the normal level, which is close to the simulation result of 2.8 times. Because the numerical simulation can not completely drilling the actual situation and the experimental process using CO and N_2_ instead of CH_4_ for experiments, the numerical results have a small gap, but the gas emission curve is consistent with each other.

Through experiments, it could be seen that the overall trend of the gas emission from the boreholes was increasing over time and the gas emission increased rapidly in the initial period. The performance of Weishe and Fenghui coal mines was very clearly evident. In the later period, it gradually slowed down, and the curve tended to be flat—the Weishe, Xuehu, and Fenghui Coal Mines were approaching 0.5, 0.5, and 1.5 L/s, respectively. When the drill bit came into contact with the soft coal, the curve experienced a sharp increase and deviated from the original trend. The maximum value of the Xuehu Coal Mine was 3 times the normal level, and the peak value reached 1.5 L/s. The peak value of Fenghui Coal Mine was as high as 5.38 L/s, which was much higher than that of the others. It was approximately 3.58 times the normal level, as shown in Fig 11.

There was some disagreement between the experimental findings and the simulation results. The starting point of the initial gas flow diagram of the borehole in the experimental results did not start from the origin of the coordinate system, unlike in the simulation curve. This was because the experiment was designed with a 60-mm coal-breaking section. In this interval, the flowmeter did not work; however, the drill bit had entered the coal seam, gas entered the sensor along the drill pipe, and some gas accumulated inside the sensor, when the test was officially started. Because the experiment used CO_2_ and N_2_ gases instead of methane, the coal had different adsorption characteristics for these three gases, and the gas resistance caused by the drilled coal chips varied with the amount of coal chips and the accumulation form; there was a small range of fluctuations in the curve; however, the overall trend was consistent with the simulation results. In the test, there was a serious downward trend in some curves. It was possible that the capsule was continuously impacted by the drill pipe during the drilling process, which reduced the sealing pressure of the capsule and caused a pressure relief. In the experiment related to the local soft coal seams, there were several obvious fluctuations in the curve, which were mainly caused by the inability of the experiment to fully reproduce the actual occurrence of the coal, the development of fissures at the location, and the uncertainty of the borehole gas resistance.

## 5 Conclusions

This research outlined in this paper investigated the law of gas emission during drilling. Through a theoretical analysis, it revealed the influence of the physical properties of the coal seams on gas emission from boreholes. Using the numerical physical coal model, showed a preliminary correlation between the drilling distance and initial gas emission was revealed.

When the drill pipe was advancing at a constant speed, by measuring the initial gas emission of the borehole from different coal samples, the relationship between the initial gas emission of the borehole and the drilling distance was obtained. The results showed that is a positive correlation between drilling depth and volume of initial gas emission in the borehole. During the drilling process, the initial gas emission increased with drilling depth, and due to the saturated adsorption capacity of coal, the tendency of gas emission rate deduced. In addition, when the drill pipe comes into contact with soft stratification, the initial gas emission surges to three times the normal level, which indicates that the initial gas emission is highly sensitive to soft stratification. Therefore, this study revealed the law of dynamic gas emission from the boreholes, and could provide a basis for gas drainage and early detection of soft coal seams.

## Supporting information

S1 Data (DOCX)
