# Supplementary material for: Numerical and experimental studies on dynamic gas emission characteristics of boreholes
Source: PLoS One. 2021 May 7;16(5):e0251209. doi: 10.1371/journal.pone.0251209 (PMC8104414; doi:10.1371/journal.pone.0251209)
Supplement: S1 Data — (DOCX) [file pone.0251209.s001.docx]

numerical simulation data

Drilling depth Initial gas emission initial gas emission initial gas emission

m L/s L/s L/s

1MPa 0.5MPa 0.1MPa

0 0 0 0

0.02222 0.09728 0.04634 0.01331

0.04444 0.19301 0.09174 0.0263

0.06667 0.28481 0.13568 0.03895

0.08889 0.3776 0.1794 0.05134

0.11111 0.4667 0.22167 0.0634

0.13333 0.5555 0.26338 0.07521

0.15556 0.64023 0.30361 0.08672

0.17778 0.72301 0.34296 0.09796

0.2 0.80532 0.38173 0.10896

0.22222 0.88698 0.41998 0.11974

0.24444 0.96743 0.45748 0.13028

0.26667 1.04248 0.49325 0.14054

0.28889 1.11755 0.5287 0.1506

0.31111 1.19377 0.56405 0.16049

0.33333 1.26837 0.59868 0.17017

0.35556 1.34002 0.63223 0.17963

0.37778 1.41228 0.66565 0.18892

0.4 1.48123 0.69793 0.198

0.42222 1.55189 0.73028 0.20693

0.44444 1.62117 0.76201 0.21569

0.46667 1.68887 0.79306 0.22427

0.48889 1.75404 0.82326 0.23267

0.51111 1.82052 0.85347 0.24093

0.53333 1.88279 0.88237 0.249

0.55556 1.9435 0.91059 0.2569

0.57778 2.00596 0.93905 0.26469

0.6 2.06199 0.96563 0.27226

0.62222 2.12053 0.99258 0.27974

0.64444 2.18006 1.01952 0.2871

0.66667 2.2382 1.04598 0.29433

0.68889 2.29156 1.07099 0.30137

0.71111 2.34915 1.09685 0.30836

0.73333 2.40267 1.12148 0.31518

0.75556 2.45348 1.14521 0.32184

0.77778 2.50509 1.16896 0.32841

0.8 2.55494 1.19208 0.33485

0.82222 2.60735 1.21562 0.34123

0.84444 2.65996 1.23906 0.34751

0.86667 2.70789 1.26113 0.35362

0.88889 2.75505 1.28287 0.35963

0.91111 2.80406 1.30489 0.36558

0.93333 2.8544 1.32705 0.37145

0.95556 2.899 1.34762 0.37715

0.97778 2.94551 1.36854 0.38279

1 2.98822 1.38831 0.38828

1.02222 3.03334 1.40853 0.39373

1.04444 3.07871 1.42864 0.3991

1.06667 3.1219 1.44808 0.40436

1.08889 3.16556 1.46753 0.40955

1.11111 3.20862 1.48667 0.41465

1.13333 3.25096 1.50547 0.41967

1.15556 3.2899 1.52328 0.42456

1.17778 3.33024 1.54132 0.4294

1.2 3.36973 1.55899 0.43415

1.22222 3.41173 1.57722 0.43888

1.24444 3.45158 1.59474 0.4435

1.26667 3.48994 1.61174 0.44803

1.28889 3.52816 1.62864 0.4525

1.31111 3.56681 1.64551 0.45691

1.33333 3.61229 1.66403 0.46136

1.35556 3.64758 1.67981 0.46558

1.37778 3.68284 1.6955 0.46975

1.4 3.71774 1.71095 0.47386

1.42222 3.75575 1.72709 0.47795

1.44444 3.79107 1.74244 0.48194

1.46667 3.82495 1.75728 0.48585

1.48889 3.85997 1.77239 0.48972

1.51111 3.89367 1.78705 0.49352

1.53333 3.93393 1.80322 0.49736

1.55556 3.96334 1.81659 0.50098

1.57778 3.99746 1.83107 0.50463

1.6 4.02844 1.84463 0.50817

1.62222 4.06222 1.85878 0.5117

1.64444 4.09586 1.87281 0.51518

1.66667 4.12647 1.88599 0.51857

1.68889 4.15666 1.89901 0.5219

1.71111 4.18644 1.91179 0.52518

1.73333 4.21722 1.92472 0.52842

1.75556 4.24504 1.93684 0.53157

1.77778 4.27561 1.94962 0.53472

1.8 4.30648 1.96236 0.53783

1.82222 4.33453 1.9743 0.54085

1.84444 4.36515 1.98679 0.54386

1.86667 4.3953 1.9991 0.54682

1.88889 4.42345 2.01086 0.54972

1.91111 4.45035 2.02216 0.55254

1.93333 4.48122 2.03445 0.55539

1.95556 4.50522 2.04488 0.55809

1.97778 4.53506 2.05674 0.56085

2 4.55789 2.06676 0.56345

2.02222 4.58655 2.07817 0.5661

2.04444 4.61469 2.08934 0.56871

2.06667 4.63911 2.09951 0.57122

2.08889 4.66606 2.11032 0.57373

2.11111 4.69204 2.12077 0.57619

2.13333 4.71805 2.13116 0.57862

2.15556 4.74076 2.14064 0.58095

2.17778 4.76639 2.15083 0.5833

2.2 4.78897 2.16014 0.58556

2.22222 4.81359 2.16991 0.58782

2.24444 4.83767 2.17945 0.59004

2.26667 4.86177 2.18896 0.59222

2.28889 4.88349 2.19785 0.59433

2.31111 4.90656 2.20697 0.59643

2.33333 4.9294 2.21598 0.59849

2.35556 4.95011 2.22436 0.60049

2.37778 4.97149 2.23291 0.60247

2.4 4.99043 2.24076 0.60438

2.42222 5.01395 2.24968 0.60632

2.44444 5.03718 2.25849 0.60823

2.46667 5.0566 2.26629 0.61005

2.48889 5.07729 2.27437 0.61186

2.51111 5.09878 2.28257 0.61365

2.53333 5.11858 2.2903 0.61539

2.55556 5.13672 2.29754 0.61707

2.57778 5.15733 2.30538 0.61876

2.6 5.1746 2.31232 0.62037

2.62222 5.19723 2.32056 0.62203

2.64444 5.21828 2.32831 0.62364

2.66667 5.23623 2.33523 0.62518

2.68889 5.25675 2.34285 0.62673

2.71111 5.27567 2.34993 0.62823

2.73333 5.29706 2.3576 0.62974

2.75556 5.31501 2.36435 0.63116

2.77778 5.33314 2.37111 0.63257

2.8 5.35028 2.37757 0.63393

2.82222 5.3683 2.3842 0.63529

2.84444 5.38843 2.39126 0.63664

2.86667 5.40632 2.39776 0.63794

2.88889 5.42242 2.40382 0.63919

2.91111 5.43987 2.41011 0.64043

2.93333 5.45875 2.4167 0.64167

2.95556 5.47421 2.4224 0.64283

2.97778 5.49157 2.42856 0.644

3 5.5072 2.43425 0.64512

3.02222 5.52385 2.44009 0.64623

3.04444 5.54253 2.44643 0.64735

3.06667 5.55718 2.45171 0.64838

3.08889 5.57137 2.45685 0.64938

3.11111 5.58959 2.46293 0.65042

3.13333 5.60867 2.46921 0.65145

3.15556 5.62662 2.47518 0.65245

3.17778 5.64424 2.48107 0.65342

3.2 5.65762 2.48585 0.6543

3.22222 5.67203 2.49076 0.65517

3.24444 5.69037 2.49663 0.65608

3.26667 5.70593 2.50178 0.65693

3.28889 5.7197 2.50648 0.65773

3.31111 5.73328 2.51105 0.65851

3.33333 5.74976 2.5163 0.65931

3.35556 5.76234 2.52053 0.66003

3.37778 5.77384 2.52448 0.66071

3.4 5.78842 2.52918 0.66142

3.42222 5.80091 2.53326 0.66208

3.44444 5.81711 2.53828 0.66277

3.46667 5.83014 2.54242 0.6634

3.48889 5.84125 2.54611 0.66398

3.51111 5.85429 2.55021 0.66457

3.53333 5.87065 2.55511 0.66519

3.55556 5.88258 2.55887 0.66572

3.57778 5.89648 2.56312 0.66627

3.6 5.90597 2.56622 0.66673

3.62222 5.91717 2.56965 0.66719

3.64444 5.92908 2.57325 0.66765

3.66667 5.94232 2.57713 0.66811

3.68889 5.95253 2.58027 0.66851

3.71111 5.96552 2.58408 0.66893

3.73333 5.9799 2.58817 0.66935

3.75556 5.98872 2.59083 0.66968

3.77778 5.99937 2.59396 0.67001

3.8 6.01161 2.59746 0.67035

3.82222 6.02268 2.60058 0.67066

3.84444 6.03613 2.60428 0.67098

3.86667 6.04419 2.60659 0.67121

3.88889 6.05501 2.60958 0.67146

3.91111 6.06305 2.61185 0.67165

3.93333 6.0748 2.61497 0.67188

3.95556 6.0832 2.61725 0.67204

3.97778 6.093 2.61985 0.67221

4 6.10189 2.62222 0.67235

Drilling depth Initial gas emission

m L/s

0 0

0.02222 0.00896

0.04444 0.01615

0.06667 0.02269

0.08889 0.03145

0.11111 0.03885

0.13333 0.05047

0.15556 0.05709

0.17778 0.06717

0.2 0.07264

0.22222 0.08105

0.24444 0.09022

0.26667 0.09632

0.28889 0.10216

0.31111 0.11087

0.33333 0.11952

0.35556 0.12429

0.37778 0.13145

0.4 0.13762

0.42222 0.1459

0.44444 0.15407

0.46667 0.15863

0.48889 0.16448

0.51111 0.17376

0.53333 0.18072

0.55556 0.18548

0.57778 0.19238

0.6 0.19534

0.62222 0.20312

0.64444 0.20989

0.66667 0.21542

0.68889 0.22081

0.71111 0.2267

0.73333 0.23481

0.75556 0.23906

0.77778 0.24309

0.8 0.24783

0.82222 0.2553

0.84444 0.26304

0.86667 0.26628

0.88889 0.2721

0.91111 0.27753

0.93333 0.28463

0.95556 0.28904

0.97778 0.29233

1 0.29623

1.02222 0.3017

1.04444 0.30825

1.06667 0.31155

1.08889 0.31554

1.11111 0.32077

1.13333 0.32621

1.15556 0.32868

1.17778 0.33412

1.2 0.33492

1.22222 0.33967

1.24444 0.34433

1.26667 0.34731

1.28889 0.35075

1.31111 0.35527

1.33333 0.35943

1.35556 0.36208

1.37778 0.36581

1.4 0.36802

1.42222 0.37225

1.44444 0.37658

1.46667 0.37946

1.48889 0.38185

1.51111 0.385

1.53333 0.38878

1.55556 0.3911

1.57778 0.39264

1.6 0.39369

1.62222 0.3973

1.64444 0.40027

1.66667 0.40273

1.68889 0.40535

1.71111 0.40742

1.73333 0.41099

1.75556 0.4112

1.77778 0.41297

1.8 0.41342

1.82222 0.41494

1.84444 0.41611

1.86667 0.41583

1.88889 0.41818

1.91111 0.41905

1.93333 0.42188

1.95556 0.42229

1.97778 0.423

2 0.42368

2.02222 0.42472

2.04444 0.42604

2.06667 0.4266

2.08889 0.4267

2.11111 0.4274

2.13333 0.42849

2.15556 0.42693

2.17778 0.42937

2.2 0.42738

2.22222 0.43238

2.24444 0.43719

2.26667 0.44166

2.28889 0.44561

2.31111 0.44982

2.33333 0.45583

2.35556 0.45999

2.37778 0.46389

2.4 0.46783

2.42222 0.47119

2.44444 0.47612

2.46667 0.47986

2.48889 0.52077

2.51111 0.54338

2.53333 0.59929

2.55556 0.64094

2.57778 0.69826

2.6 0.73981

2.62222 0.78309

2.64444 0.844

2.66667 0.8803

2.68889 0.91988

2.71111 0.96715

2.73333 1.01446

2.75556 1.06093

2.77778 1.09578

2.8 1.13982

2.82222 1.18938

2.84444 1.23427

2.86667 1.28272

2.88889 1.32557

2.91111 1.37592

2.93333 1.43424

2.95556 1.474

2.97778 1.51478

3 1.55669

3.02222 1.60892

3.04444 1.6759

3.06667 1.72793

3.08889 1.76637

3.11111 1.81517

3.13333 1.86143

3.15556 1.90472

3.17778 1.94741

3.2 1.99113

3.22222 2.03058

3.24444 2.08253

3.26667 2.13237

3.28889 2.02685

3.31111 1.884

3.33333 1.74114

3.35556 1.59828

3.37778 1.45543

3.4 1.31257

3.42222 1.16971

3.44444 1.02685

3.46667 0.884

3.48889 0.74114

3.51111 0.78263

3.53333 0.7889

3.55556 0.79343

3.57778 0.79834

3.6 0.80239

3.62222 0.80689

3.64444 0.81366

3.66667 0.81771

3.68889 0.822

3.71111 0.82627

3.73333 0.83385

3.75556 0.83944

3.77778 0.84315

3.8 0.84833

3.82222 0.85385

3.84444 0.85933

3.86667 0.86315

3.88889 0.86717

3.91111 0.87155

3.93333 0.87709

3.95556 0.88066

3.97778 0.88639

4 0.89075

experimental data

Wuzhong

0.5 0.127496956

1 0.121420269

1.5 0.126392184

2 0.147937003

2.5 0.172797024

3 0.173901796

3.5 0.155118905

4 0.135231244

4.5 0.114790753

5 0.104846922

5.5 0.072846755

6 0.103189764

6.5 0.104846922

7 0.102084993

7.5 0.071441834

8 0.069334545

8.5 0.068163934

9 0.068398056

9.5 0.109266451

10 0.139650773

10.5 0.162853193

11 0.162300364

11.5 0.170034652

12 0.188265156

12.5 0.194894229

13 0.182740855

13.5 0.183293241

14 0.19655183

14.5 0.17776894

15 0.161195592

15.5 0.167272723

16 0.169482266

16.5 0.166719894

17 0.172797024

17.5 0.194894229

18 0.192684686

18.5 0.176111339

19 0.198208987

19.5 0.210362805

20 0.182740855

20.5 0.190474699

21 0.212572348

21.5 0.20097136

22 0.192684686

22.5 0.220306636

23 0.236879539

23.5 0.219201864

24 0.235222382

24.5 0.260634345

25 0.24682337

25.5 0.21533472

26 0.241851455

26.5 0.245166213

27 0.211467576

27.5 0.225830937

28 0.244061441

28.5 0.230250466

29 0.230250466

29.5 0.254557658

30 0.246270984

30.5 0.24240384

31 0.26560626

31.5 0.275550535

32 0.250138128

32.5 0.263949103

33 0.29322821

33.5 0.292675824

34 0.281074836

34.5 0.311458714

35 0.341843036

35.5 0.344052579

36 0.365597842

36.5 0.381065974

37 0.331346376

37.5 0.307039185

38 0.332451591

38.5 0.310353943

39 0.27002579

39.5 0.303724426

40 0.339080664

40.5 0.336318291

41 0.344604965

41.5 0.381065974

42 0.378855987

42.5 0.336318291

43 0.360073097

43.5 0.388247432

44 0.379961203

44.5 0.389352648

45 0.420841297

45.5 0.424156056

46 0.392114576

46.5 0.395429335

47 0.421393683

47.5 0.405925551

48 0.393219348

48.5 0.427470814

49 0.44680609

49.5 0.432995115

50 0.441281789

50.5 0.458959464

51 0.45122562

51.5 0.42360367

52 0.435757487

52.5 0.455645149

53 0.455092763

53.5 0.452330391

54 0.477742354

54.5 0.48437187

55 0.458959464

55.5 0.448463247

56 0.474428039

56.5 0.475532811

57 0.462826608

57.5 0.475532811

58 0.486581413

58.5 0.458959464

59 0.429680357

59.5 0.456197535

60 0.462274222

60.5 0.430232743

61 0.436309873

61.5 0.46116945

62 0.46116945

62.5 0.430232743

63 0.454539934

63.5 0.478847569

64 0.452330391

64.5 0.427470814

65 0.466141366

65.5 0.486029028

66 0.468350909

66.5 0.455645149

67 0.493763315

67.5 0.506469519

68 0.463378993

68.5 0.447358476

69 0.473322824

69.5 0.467246137

70 0.43796703

70.5 0.457854692

71 0.480504726

71.5 0.462274222

72 0.455645149

72.5 0.494868087

73 0.505916689

73.5 0.477742354

74 0.494315701

74.5 0.518622893

75 0.504259532

75.5 0.494315701

76 0.519727664

76.5 0.519727664

77 0.472218053

77.5 0.466141366

78 0.4887914

78.5 0.459512293

79 0.437414645

79.5 0.477189968

80 0.492658544

80.5 0.466141366

81 0.485476642

81.5 0.521937651

82 0.492658544

82.5 0.463931379

83 0.497630459

83.5 0.50149716

84 0.456749921

84.5 0.464484209

85 0.505364304

85.5 0.486581413

86 0.461721836

86.5 0.493763315

87 0.512546206

87.5 0.494315701

88 0.497078074

88.5 0.53519624

89 0.528014338

89.5 0.498735231

90 0.523042422

90.5 0.555083902

91 0.530224324

91.5 0.509231447

92 0.542377698

92.5 0.552873915

93 0.455092763

93.5 0.387142661

94 0.417526983

94.5 0.398191707

95 0.281074836

95.5 0.27444532

96 0.342395422

96.5 0.320850159

97 0.296542968

97.5 0.370569757

98 0.419736526

98.5 0.340737821

99 0.30759157

99.5 0.361730698

100 0.370017372

100.5 0.361730698

101 0.427470814

101.5 0.464484209

102 0.378855987

102.5 0.333556363

103 0.363387855

103.5 0.332451591

104 0.297095354

104.5 0.359520711

105 0.399296479

105.5 0.361730698

106 0.393219348

106.5 0.467246137

107 0.494315701

107.5 0.483267099

108 0.463378993

108.5 0.432442729

109 0.420288912

109.5 0.456197535

110 0.521937651

110.5 0.541825313

111 0.56668489

111.5 0.643474052

112 0.677172688

112.5 0.639054522

113 0.638502136

113.5 0.616404488

114 0.531881482

114.5 0.471665667

115 0.4887914

115.5 0.456197535

116 0.389905033

116.5 0.433547501

117 0.466693752

117.5 0.430785128

118 0.453987548

118.5 0.510888605

119 0.487686628

119.5 0.483267099

120 0.529119109

120.5 0.546244842

121 0.532986253

121.5 0.567237275

122 0.581600636

122.5 0.510336219

123 0.510336219

123.5 0.551769143

124 0.53519624

124.5 0.521385265

125 0.580495864

125.5 0.592649682

126 0.525251965

126.5 0.524147194

127 0.555636287

127.5 0.526909566

128 0.531881482

128.5 0.582705851

129 0.560608203

129.5 0.521937651

130 0.568894876

130.5 0.587125381

131 0.562817746

131.5 0.593754454

132 0.626348319

132.5 0.57718155

133 0.542930084

133.5 0.579943479

134 0.580495864

134.5 0.531881482

135 0.560055817

135.5 0.59983114

136 0.56668489

136.5 0.574971563

137 0.640711679

137.5 0.642368836

138 0.600383526

138.5 0.631320234

139 0.657837412

139.5 0.635187378

140 0.666123642

140.5 0.695955578

141 0.645131209

141.5 0.625795933

142 0.640159293

142.5 0.603698284

143 0.587125381

143.5 0.640711679

144 0.610880187

144.5 0.572209191

145 0.624691161

145.5 0.620824017

146 0.572209191

146.5 0.615852102

147 0.629663077

147.5 0.540720541

148 0.537405783

148.5 0.588230152

149 0.558951045

149.5 0.546244842

150 0.619166416

150.5 0.618061645

151 0.556741059

151.5 0.582153465

152 0.62303356

152.5 0.611984958

153 0.629663077

153.5 0.685458918

154 0.664466485

154.5 0.624691161

155 0.653417882

155.5 0.670543172

156 0.642368836

156.5 0.64623598

157 0.682696989

157.5 0.654522654

158 0.609774971

158.5 0.639606908

159 0.648998353

159.5 0.6086702

160 0.629663077

160.5 0.6694384

161 0.628005476

161.5 0.596516826

162 0.641816451

162.5 0.646788366

163 0.611984958

163.5 0.651207896

164 0.683249375

164.5 0.630767848

165 0.603145899

165.5 0.659494569

166 0.655627425

166.5 0.603145899

167 0.62745309

167.5 0.654522654

168 0.582153465

168.5 0.548454385

169 0.602593513

169.5 0.610880187

170 0.567237275

170.5 0.617509259

171 0.657284583

171.5 0.573314406

172 0.53519624

172.5 0.593202068

173 0.607013043

173.5 0.558951045

174 0.584363008

174.5 0.658389798

175 0.626900704

175.5 0.542930084

176 0.568342491

176.5 0.632977391

177 0.623585946

177.5 0.604803056

178 0.666123642

178.5 0.688773676

179 0.63187262

179.5 0.616404488

180 0.661704112

180.5 0.665018871

181 0.661151727

181.5 0.705347023

Weishe

0.5 0.14572746

1 0.139650773

1.5 0.133573643

2 0.12694457

2.5 0.120315497

3 0.108714066

3.5 0.072612633

4 0.071441834

4.5 0.10374215

5 0.110371223

5.5 0.108714066

6 0.110924052

6.5 0.12252504

7 0.125839799

7.5 0.121972655

8 0.120867883

8.5 0.127496956

9 0.126392184

9.5 0.130259328

10 0.136336015

10.5 0.129154113

11 0.129154113

11.5 0.140203159

12 0.138545558

12.5 0.132468871

13 0.149042219

13.5 0.153461748

14 0.147937003

14.5 0.166719894

15 0.176664168

15.5 0.156223677

16 0.163405579

16.5 0.176111339

17 0.163405579

17.5 0.153461748

18 0.167272723

18.5 0.160090821

19 0.137440787

19.5 0.139650773

20 0.147384618

20.5 0.139098388

21 0.138545558

21.5 0.158433664

22 0.154014134

22.5 0.135231244

23 0.14572746

23.5 0.165062736

24 0.160643207

24.5 0.159538435

25 0.181083254

25.5 0.185502784

26 0.167272723

26.5 0.185502784

27 0.207048046

27.5 0.198761373

28 0.194894229

28.5 0.214782334

29 0.215887106

29.5 0.204838503

30 0.208705204

30.5 0.215887106

31 0.209257589

31.5 0.212019962

32 0.226935708

32.5 0.221411407

33 0.208705204

33.5 0.226383322

34 0.236879539

34.5 0.223621394

35 0.227488094

35.5 0.245166213

36 0.24295667

36.5 0.233012395

37 0.248480971

37.5 0.255662429

38 0.237431925

38.5 0.235774768

39 0.250690514

39.5 0.244613827

40 0.224726165

40.5 0.24240384

41 0.251795285

41.5 0.237431925

42 0.244613827

42.5 0.261186731

43 0.259529573

43.5 0.255110044

44 0.273340548

44.5 0.27444532

45 0.26173956

45.5 0.277207692

46 0.286599137

46.5 0.269473404

47 0.271683391

47.5 0.291571053

48 0.293780596

48.5 0.282179607

49 0.293780596

49.5 0.308696786

50 0.301514883

50.5 0.295438196

51 0.313115872

51.5 0.306486799

52 0.278312463

52.5 0.291018667

53 0.302619655

53.5 0.279417235

54 0.26173956

54.5 0.286599137

55 0.28880868

55.5 0.256767645

56 0.260634345

56.5 0.28880868

57 0.279417235

57.5 0.263396717

58 0.293780596

58.5 0.30759157

59 0.279417235

59.5 0.267816247

60 0.286046751

60.5 0.277207692

61 0.24682337

61.5 0.254557658

62 0.278312463

62.5 0.258424802

63 0.239641912

63.5 0.271683391

64 0.278312463

64.5 0.261186731

65 0.279417235

65.5 0.298200125

66 0.293780596

66.5 0.295990582

67 0.319193002

67.5 0.309801557

68 0.294333425

68.5 0.320850159

69 0.321954931

69.5 0.3120111

70 0.324717303

70.5 0.334661134

71 0.324717303

71.5 0.340737821

72 0.340737821

72.5 0.318088231

73 0.320850159

73.5 0.324717303

74 0.29930534

74.5 0.294885811

75 0.317535401

75.5 0.316983015

76 0.302067269

76.5 0.315325858

77 0.319745388

77.5 0.306486799

78 0.323612532

78.5 0.331346376

79 0.298200125

79.5 0.300410112

80 0.323612532

80.5 0.301514883

81 0.282179607

81.5 0.314773472

82 0.326926846

82.5 0.308143956

83 0.324164918

83.5 0.354548796

84 0.334661134

84.5 0.323060146

85 0.358968326

85.5 0.361178312

86 0.325269689

86.5 0.338528278

87 0.3727793

87.5 0.361730698

88 0.342947808

88.5 0.370569757

89 0.371122143

89.5 0.339080664

90 0.355654011

90.5 0.380513588

91 0.361178312

91.5 0.350681652

92 0.387695047

92.5 0.386037889

93 0.347919723

93.5 0.360073097

94 0.382723131

94.5 0.363940241

95 0.354548796

95.5 0.389905033

96 0.405925551

96.5 0.376094059

97 0.379961203

97.5 0.414764611

98 0.397086492

98.5 0.357311168

99 0.377751216

99.5 0.401506022

100 0.371674529

100.5 0.349024495

101 0.386590275

101.5 0.394876949

102 0.371674529

102.5 0.38161836

103 0.417526983

103.5 0.420841297

104 0.387142661

104.5 0.396534106

105 0.424156056

105.5 0.408687924

106 0.386037889

106.5 0.416421768

107 0.431337958

107.5 0.389352648

108 0.356758783

108.5 0.384380732

109 0.402058407

109.5 0.379961203

110 0.380513588

110.5 0.422498898

111 0.411449852

111.5 0.373884072

112 0.394324563

112.5 0.426366042

113 0.417526983

113.5 0.409240309

114 0.442938946

114.5 0.448463247

115 0.407030323

115.5 0.397638878

116 0.428575585

116.5 0.421946513

117 0.397638878

117.5 0.416421768

118 0.438519416

118.5 0.426918428

119 0.421946513

119.5 0.456749921

120 0.458959464

120.5 0.418631754

121 0.413107453

121.5 0.440729403

122 0.428575585

122.5 0.398191707

123 0.419736526

123.5 0.437414645

124 0.418079369

124.5 0.411449852

125 0.44680609

125.5 0.450120848

126 0.413107453

126.5 0.422498898

127 0.454539934

127.5 0.440729403

128 0.413107453

128.5 0.435757487

129 0.453435163

129.5 0.418631754

130 0.402058407

130.5 0.441834174

131 0.459512293

131.5 0.422498898

132 0.398191707

132.5 0.433547501

133 0.445701318

133.5 0.408687924

134 0.402058407

134.5 0.435757487

135 0.414212225

135.5 0.388247432

136 0.425813213

136.5 0.441281789

137 0.4280232

137.5 0.445701318

138 0.469456124

138.5 0.432995115

139 0.402610793

139.5 0.441281789

140 0.435757487

140.5 0.382170746

141 0.398744093

141.5 0.424708441

142 0.373884072

142.5 0.344604965

143 0.398191707

143.5 0.418079369

144 0.370569757

144.5 0.363940241

145 0.429127971

145.5 0.455645149

146 0.413107453

146.5 0.415316996

147 0.450120848

147.5 0.432995115

148 0.407030323

148.5 0.439624188

149 0.44680609

149.5 0.401506022

150 0.409240309

150.5 0.436309873

151 0.4280232

151.5 0.410345081

152 0.440729403

152.5 0.451778005

153 0.422498898

153.5 0.430785128

154 0.47000851

154.5 0.471113281

155 0.435757487

155.5 0.44680609

156 0.479952341

156.5 0.460617065

157 0.416421768

157.5 0.437414645

158 0.46116945

158.5 0.426918428

159 0.415316996

159.5 0.445701318

160 0.432442729

160.5 0.398191707

161 0.436862259

161.5 0.457854692

162 0.426366042

162.5 0.394876949

163 0.425260827

163.5 0.430232743

164 0.384380732

164.5 0.382723131

165 0.426918428

165.5 0.431890344

166 0.397086492

166.5 0.408687924

167 0.432995115

167.5 0.412555068

168 0.3727793

168.5 0.399848864

169 0.408135538

169.5 0.357863554

170 0.344604965

170.5 0.356206397

171 0.320850159

171.5 0.33853714

172 0.329145695

172.5 0.344613827

173 0.313124733

173.5 0.275558953

174 0.309257589

174.5 0.326935708

175 0.295999444

175.5 0.29655183

176 0.337984754

176.5 0.339641912

177 0.314229949

177.5 0.298424802

178 0.327479676

178.5 0.33079399

179 0.319745388

179.5 0.389352648

180 0.436862259

180.5 0.40040125

181 0.394324563

181.5 0.456197535

182 0.470560896

182.5 0.413659839

183 0.405373165

183.5 0.455645149

184 0.465036594

184.5 0.402058407

185 0.399848864

185.5 0.444596103

186 0.447358476

186.5 0.392114576

187 0.412002238

187.5 0.461721836

188 0.46116945

188.5 0.400953636

189 0.400953636

189.5 0.44238656

190 0.461721836

190.5 0.451778005

191 0.481609498

191.5 0.498182845

192 0.460064679

192.5 0.432995115

193 0.46116945

193.5 0.471665667

194 0.436309873

194.5 0.414764611

195 0.448463247

195.5 0.463931379

196 0.431337958

196.5 0.405373165

197 0.446253704

197.5 0.485476642

198 0.475532811

198.5 0.452882777

Xuehu

4 0.278389354

4.5 0.255910287

5 0.213970543

5.5 0.222310833

6 0.207956456

6.5 0.51407735

7 0.434681489

7.5 0.380568221

8 0.356173006

8.5 0.355729496

9 0.360164957

9.5 0.385891059

10 0.404076415

10.5 0.421375106

11 0.434237979

11.5 0.439117307

12 0.439117307

12.5 0.438229929

13 0.435125356

13.5 0.447101208

14 0.474157842

14.5 0.489682135

15 0.46883536

15.5 0.405850813

16 0.306495199

16.5 0.187179475

17 0.08693684

17.5 0.036052331

18 0.033133057

18.5 0.035898698

19 0.055000878

19.5 0.092259322

20 0.135283759

20.5 0.1663327

21 0.183631035

21.5 0.190727915

22 0.200486215

22.5 0.213792599

23 0.220889479

23.5 0.21867157

24 0.220445969

24.5 0.223107388

25 0.223994409

25.5 0.220002458

26 0.216897528

26.5 0.219558948

27 0.22133299

27.5 0.220002458

28 0.21867157

28.5 0.21512313

29 0.21512313

29.5 0.210244158

30 0.207582739

30.5 0.220445969

31 0.259921966

31.5 0.30383378

32 0.332220945

32.5 0.343753287

33 0.337543784

33.5 0.327341974

34 0.317140164

34.5 0.310043639

35 0.306495199

35.5 0.308712752

36 0.308712752

36.5 0.30427729

37 0.291414417

37.5 0.288309487

38 0.286535089

38.5 0.283874026

39 0.279882075

39.5 0.281212607

40 0.283874026

40.5 0.281656117

41 0.277220656

41.5 0.279882075

42 0.284317537

42.5 0.284761047

43 0.285204558

43.5 0.280325586

44 0.278994698

44.5 0.279438209

45 0.279882075

45.5 0.283430516

46 0.284317537

46.5 0.283874026

47 0.283874026

47.5 0.285648068

48 0.286535089

48.5 0.286535089

49 0.282543138

49.5 0.282543138

50 0.279882075

50.5 0.279438209

51 0.278994698

51.5 0.278994698

52 0.272341684

52.5 0.270123776

53 0.269680265

53.5 0.266131825

54 0.262583385

54.5 0.259034945

55 0.260809343

55.5 0.261696364

56 0.256817036

56.5 0.252381575

57 0.247059092

57.5 0.240849233

58 0.212018557

58.5 0.164558302

59 0.145042059

59.5 0.14903401

60 0.158792309

60.5 0.168106742

61 0.174316602

61.5 0.190284405

62 0.208026606

62.5 0.216897528

63 0.214236109

63.5 0.209800648

64 0.204478166

64.5 0.203590789

65 0.220002458

65.5 0.278107677

66 0.326898463

66.5 0.364156908

67 0.385891059

67.5 0.374802228

68 0.29762392

68.5 0.281656117

69 0.341092224

69.5 0.394761982

70 0.421375106

70.5 0.424923546

71 0.349076126

71.5 0.281656117

72 0.334882365

72.5 0.404520282

73 0.447544719

73.5 0.468391493

74 0.475488374

74.5 0.37657627

75 0.23508324

75.5 0.232865331

76 0.330003393

76.5 0.394318471

77 0.422262127

77.5 0.418270176

78 0.332664812

78.5 0.22532494

79 0.274559237

79.5 0.362826376

80 0.358834069

80.5 0.281212607

81 0.14592908

81.5 0.07008166

82 0.175647133

82.5 0.268793244

83 0.263913916

83.5 0.23463973

84 0.161009862

84.5 0.090041414

85 0.144155038

85.5 0.264357427

86 0.275446258

86.5 0.253268952

87 0.197381285

87.5 0.113106453

88 0.110888544

88.5 0.208913627

89 0.242180121

89.5 0.219115437

90 0.184961923

90.5 0.13927571

91 0.122864397

91.5 0.222663521

92 0.291414417

92.5 0.22842987

93 0.130848298

93.5 0.058992829

94 0.050121906

94.5 0.074073611

95 0.077622051

95.5 0.067420241

96 0.049678396

96.5 0.03789605

97 0.038589564

97.5 0.087380351

98 0.181413482

98.5 0.290083529

99 0.39343145

99.5 0.502101497

100 0.744628586

100.5 0.589215509

101 0.532705789

101.5 0.418713686

102 0.372140809

102.5 0.437786419

103 0.510085399

103.5 0.518578359

104 0.518578359

104.5 0.432907447

105 0.257704413

105.5 0.214236109

106 0.244397673

106.5 0.271454307

107 0.2869786

107.5 0.289640019

108 0.302946759

108.5 0.334438854

109 0.375245739

109.5 0.415608757

110 0.45863355

110.5 0.493674085

111 0.514521216

111.5 0.529158132

112 0.518578359

112.5 0.476184721

113 0.518578359

113.5 0.534924481

114 0.522061607

114.5 0.506536959

115 0.489238624

115.5 0.473714332

116 0.459520571

116.5 0.449762271

117 0.447101208

117.5 0.443552768

118 0.453754578

118.5 0.477706283

119 0.436012377

119.5 0.352624566

120 0.274559237

120.5 0.252825085

121 0.266575335

121.5 0.298954808

122 0.329116372

122.5 0.341979245

123 0.345971196

123.5 0.349076126

124 0.387665101

124.5 0.443109257

125 0.471052913

125.5 0.44843174

126 0.361495488

126.5 0.375245739

127 0.460851102

127.5 0.52472267

128 0.603342939

128.5 0.546833219

129 0.471939934

129.5 0.502545008

130 0.560960649

130.5 0.645736576

131 0.673991436

131.5 0.53403746

132 0.293188459

132.5 0.161896883

133 0.259478455

133.5 0.403632904

134 0.520287209

134.5 0.688118866

135 0.942435301

135.5 0.772894793

136 0.490312151

136.5 0.645736576

137 0.758767363

137.5 0.843531943

138 0.956562731

138.5 0.998956368

139 1.041338658

139.5 1.140242015

140 1.196751735

140.5 1.196751735

141 1.253272803

141.5 1.38043102

142 1.45106817

142.5 1.507589237

143 1.507589237

143.5 1.225006595

144 0.730501156

144.5 0.264250577

145 0.066455209

145.5 0.482141744

146 0.422705637

146.5 0.356616517

147 0.293188459

147.5 0.23863168

148 0.196494264

148.5 0.184074545

149 0.209357137

149.5 0.268793244

150 0.370366411

150.5 0.440891349

151 0.328228995

151.5 0.161896883

152 0.039476585

152.5 0.011468706

153 0.02237776

153.5 0.087823861

154 0.178752063

154.5 0.258147924

155 0.31093066

155.5 0.345971196

156 0.359721446

156.5 0.354842118

157 0.335325875

157.5 0.317140164

158 0.298067431

158.5 0.282543138

159 0.269236755

159.5 0.257704413

160 0.245284694

160.5 0.230647779

161 0.216897528

161.5 0.209800648

162 0.211575046

162.5 0.212462067

163 0.21467962

163.5 0.216897528

164 0.225768451

164.5 0.274559237

165 0.317140164

165.5 0.30693871

166 0.269680265

166.5 0.243067142

167 0.252825085

167.5 0.301615871

168 0.340648714

168.5 0.340648714

169 0.326898463

169.5 0.302059382

170 0.280325586

170.5 0.271010797

171 0.264357427

171.5 0.256817036

172 0.251051043

172.5 0.249277001

173 0.248389624

173.5 0.243067142

174 0.23463973

174.5 0.23197831

175 0.23197831

175.5 0.232421821

176 0.229760402

176.5 0.226211961

177 0.220002458

177.5 0.219115437

178 0.216010507

178.5 0.212905578

179 0.207139229

179.5 0.204478166

180 0.204921676

180.5 0.204478166

181 0.202703768

181.5 0.200042348

182 0.200042348

182.5 0.200929726

183 0.202703768

183.5 0.199598838

184 0.194276356

184.5 0.190727915

185 0.191171426

185.5 0.193832845

186 0.195163377

186.5 0.196050398

187 0.197381285

187.5 0.200929726

188 0.203147278

188.5 0.202260257

189 0.201373236

189.5 0.200929726

190 0.199598838

190.5 0.199155327

191 0.193832845

191.5 0.188066496

192 0.186292454

192.5 0.185848944

193 0.183631035

193.5 0.180969616

194 0.176977665

194.5 0.174316602

195 0.172985714

195.5 0.169881141

196 0.169437274

196.5 0.167663232

197 0.165888834

197.5 0.163227771

198 0.161009862

198.5 0.158348443

199 0.156574401

199.5 0.157461422

200 0.157461422

Huifeng

0.5 0.213124733

1 0.201523745

1.5 0.194894229

2 0.194341843

2.5 0.197656601

3 0.197104216

3.5 0.203180903

4 0.199313759

4.5 0.199313759

5 0.200418974

5.5 0.191027529

6 0.188265156

6.5 0.214229949

7 0.267816247

7.5 0.303724426

8 0.314221087

8.5 0.319745388

9 0.317535401

9.5 0.309249171

10 0.298752511

10.5 0.325822075

11 0.371122143

11.5 0.397638878

12 0.418631754

12.5 0.458407078

13 0.478847569

13.5 0.482714269

14 0.498182845

14.5 0.499840002

15 0.482161884

15.5 0.495972858

16 0.489896171

16.5 0.455645149

17 0.474428039

17.5 0.497630459

18 0.479952341

18.5 0.483267099

19 0.505916689

19.5 0.462826608

20 0.441281789

20.5 0.460617065

21 0.455645149

21.5 0.463931379

22 0.504811918

22.5 0.509783833

23 0.502049989

23.5 0.533538639

24 0.524147194

24.5 0.479399955

25 0.497630459

25.5 0.525804795

26 0.521385265

26.5 0.551216758

27 0.586572551

27.5 0.579943479

28 0.58988731

28.5 0.619719246

29 0.58104825

29.5 0.548454385

30 0.583810623

30.5 0.592097296

31 0.582705851

31.5 0.620271632

32 0.614746887

32.5 0.574971563

33 0.602041127

33.5 0.639054522

34 0.641264065

34.5 0.673305544

35 0.715290854

35.5 0.713633253

36 0.726327825

36.5 0.767942573

37 0.726327825

37.5 0.688773676

38 0.692088434

38.5 0.713080868

39 0.698165121

39.5 0.684713078

40 0.75407099

40.5 0.690983663

41 0.692088434

41.5 0.767942573

42 0.712456243

42.5 0.740199408

43 0.79569688

43.5 0.781814155

44 0.698717507

44.5 0.726327825

45 0.740199408

45.5 0.69858466

46 0.781814155

46.5 0.837311627

47 0.726327825

47.5 0.740199408

48 0.767942573

48.5 0.767942573

49 0.809568462

49.5 0.823440045

50 0.767942573

50.5 0.781814155

51 0.823440045

51.5 0.837311627

52 0.892797957

52.5 0.920552264

53 0.823440045

53.5 0.823440045

54 0.809568462

54.5 0.809568462

55 0.809568462

55.5 0.865054792

56 0.823440045

56.5 0.823440045

57 0.90666954

57.5 0.85118321

58 0.865054792

58.5 0.878926375

59 0.809568462

59.5 0.69858466

60 0.740199408

60.5 0.726327825

61 0.75407099

61.5 0.90666954

62 0.934423846

62.5 0.865054792

63 0.79569688

63.5 0.823440045

64 0.75407099

64.5 0.781814155

65 0.878926375

65.5 0.837311627

66 0.837311627

66.5 0.892797957

67 0.865054792

67.5 0.837311627

68 0.90666954

68.5 0.90666954

69 0.878926375

69.5 0.989910177

70 1.017653342

70.5 1.045407648

71 1.114765561

71.5 1.170263033

72 1.142508726

72.5 1.142508726

73 1.170263033

73.5 1.21187778

74 1.433845384

74.5 1.225749363

75 1.142508726

75.5 1.087022396

76 1.003781759

76.5 0.962167012

77 0.865054792

77.5 0.90666954

78 0.865054792

78.5 0.85118321

79 0.90666954

79.5 0.878926375

80 0.865054792

80.5 0.892797957

81 0.837311627

81.5 0.809568462

82 0.823440045

82.5 0.781814155

83 0.740199408

83.5 0.781814155

84 0.781814155

84.5 0.69858466

85 0.75407099

85.5 0.740199408

86 0.726327825

86.5 0.767942573

87 0.79569688

87.5 0.712456243

88 0.726327825

88.5 0.767942573

89 0.85118321

89.5 0.892797957

90 0.948295429

90.5 0.948295429

91 0.920552264

91.5 0.962167012

92 1.017653342

92.5 0.976038594

93 1.003781759

93.5 1.017653342

94 0.948295429

94.5 0.962167012

95 1.003781759

95.5 1.087022396

96 1.15639145

96.5 1.281246835

97 1.572572351

97.5 2.196849272

98 2.626912897

98.5 2.626912897

99 2.557543843

99.5 2.48818593

100 2.12749136

100.5 1.919395339

101 1.836154702

101.5 1.919395339

102 2.335587381

102.5 2.94599272

103 2.973735885

103.5 2.696281952

104 2.474314348

104.5 2.293961492

105 2.113619777

105.5 1.919395339

106 1.7251709

106.5 1.544829186

107 1.419973801

107.5 1.239620945

108 1.003781759

108.5 1.059279231

109 1.059279231

109.5 1.031536066

110 1.114765561

110.5 1.184134615

111 1.253492528

111.5 1.281246835

112 1.30899

112.5 1.322861582

113 1.350604747

113.5 1.350604747

114 1.253492528

114.5 1.281246835

115 1.36447633

115.5 1.281246835

116 1.336733165

116.5 1.392230636

117 1.433845384

117.5 1.350604747

118 1.433845384

118.5 1.378347912

119 1.322861582

119.5 1.36447633

120 1.433845384

120.5 1.36447633

121 1.36447633

121.5 1.392230636

122 1.503214438

122.5 1.7251709

123 1.947138504

123.5 2.016507558

124 1.752925207

124.5 1.586443933

125 1.572572351

125.5 1.544829186

126 1.419973801

126.5 1.489331714

127 1.586443933

127.5 1.558700768

128 1.628069823

128.5 1.766796789

129 1.739053624

129.5 1.614187098

130 1.7251709

130.5 1.711299318

131 1.586443933

131.5 1.586443933

132 1.655812988

132.5 1.586443933

133 1.378347912

133.5 1.350604747

134 1.406102219

134.5 1.36447633

135 1.336733165

135.5 1.392230636

136 1.392230636

136.5 1.253492528

137 1.253492528

137.5 1.322861582

138 1.281246835

138.5 1.198006198

139 1.239620945

139.5 1.253492528

140 1.184134615

140.5 1.170263033

141 1.21187778

141.5 1.198006198

142 1.322861582

142.5 1.142508726

143 1.198006198

143.5 1.100893978

144 1.059279231

144.5 1.031536066

145 1.350604747

145.5 1.087022396

146 1.003781759

146.5 1.059279231

147 1.295118417

147.5 1.059279231

148 1.073150813

148.5 1.350604747

149 1.114765561

149.5 1.031536066

150 1.045407648

150.5 1.100893978

151 1.045407648

151.5 1.059279231

152 1.100893978

152.5 1.031536066

153 0.976038594

153.5 0.989910177

154 1.003781759

154.5 0.948295429

155 0.962167012

155.5 1.017653342

156 0.948295429

156.5 0.892797957

157 0.962167012

157.5 0.962167012

158 0.934423846

158.5 0.920552264

159 1.017653342

159.5 1.100893978

160 1.100893978

160.5 1.198006198

161 1.225749363

161.5 1.253492528

162 1.350604747

162.5 1.350604747

163 1.336733165

163.5 1.433845384

164 1.461588549

164.5 1.655812988

165 1.988764393

165.5 2.252346744

166 2.169106107

166.5 2.099748195

167 2.141362942

167.5 2.196849272

168 2.391073711

168.5 2.724025117

169 2.710153534

169.5 2.474314348

170 2.446560041

170.5 2.502057513

171 2.335587381

171.5 2.169106107

172 2.085865471

172.5 1.919395339

173 1.7251709

173.5 1.641941405

174 1.600315516

174.5 1.517086021

175 1.447716966

175.5 1.447716966

176 1.406102219

176.5 1.336733165

177 1.322861582

177.5 1.378347912

178 1.378347912

178.5 1.392230636

179 1.461588549

179.5 1.558700768

180 1.628069823

180.5 1.697427735

181 1.739053624

181.5 1.739053624

182 1.822283119

182.5 1.961010086

183 2.224603579

183.5 2.58529815

184 2.890495248

184.5 3.431542675

185 3.556398059

185.5 3.875477882

186 4.180674981

186.5 4.166803399

187 3.417671093

187.5 3.056976522

188 3.91709263

188.5 5.124031726

189 5.387614077

189.5 4.818834627

190 3.695125026

190.5 2.48818593

191 1.544829186

191.5 1.045407648

192 0.90666954

192.5 0.878926375

193 0.989910177

193.5 1.087022396

194 1.184134615

194.5 1.253492528

195 1.281246835

195.5 1.350604747

196 1.392230636

196.5 1.406102219

197 1.36447633

197.5 1.322861582

198 1.30899

198.5 1.30899

199 1.30899

199.5 1.295118417

200 1.295118417

200.5 1.295118417

201 1.225749363

201.5 1.253492528

202 1.225749363

202.5 1.239620945

203 1.253492528

203.5 1.267375252

204 1.281246835

204.5 1.295118417

205 1.30899

205.5 1.295118417

206 1.295118417

206.5 1.517086021

207 1.281246835

207.5 1.267375252

208 1.15639145

208.5 1.100893978

209 1.073150813

209.5 1.087022396

210 1.128637143

210.5 1.184134615

211 1.253492528

211.5 1.30899

212 1.350604747

212.5 1.392230636

213 1.406102219

213.5 1.406102219

214 1.350604747

214.5 1.336733165

215 1.30899

215.5 1.295118417

216 1.295118417

216.5 1.253492528

217 1.267375252

217.5 1.253492528

218 1.198006198

218.5 1.170263033

219 1.184134615

219.5 1.198006198

220 1.15639145

220.5 1.15639145

221 1.184134615

221.5 1.170263033

222 1.128637143

222.5 1.128637143

223 1.142508726

223.5 1.100893978

224 1.087022396
